# A Low-Order Permutationally Invariant Polynomial Approach to Learning Potential Energy Surfaces Using the Bond-Order Charge-Density Matrix: Application to C_*n*_ Clusters for *n* = 3–10, 20

**DOI:** 10.1021/acs.jpca.4c04281

**Published:** 2024-08-29

**Authors:** Jose Gutierrez-Cardenas, Benjamin D. Gibbas, Kyle Whitaker, Martina Kaledin, Alexey L. Kaledin

**Affiliations:** † Department of Chemistry & Biochemistry, Kennesaw State University, 370 Paulding Ave NW ,Box#1203,Kennesaw 30144, Georgia; ‡ Cherry L. Emerson Center for Scientific Computation and Department of Chemistry, Emory University, 1515 Dickey Drive ,Atlanta 30322, Georgia

## Abstract

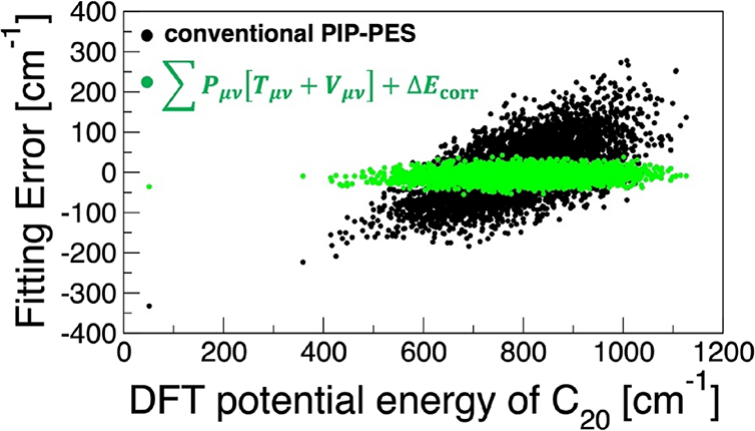

A representation for learning potential energy surfaces (PESs) in terms of permutationally invariant polynomials (PIPs) using the Hartree–Fock expression for electronic energy is proposed. Our approach is based on the one-electron core Hamiltonian weighted by the configuration-dependent elements of the bond-order charge density matrix (CDM). While the previously reported model used an *s*-function Gaussian basis for the CDM, the present formulation is expanded with *p*-functions, which are crucial for describing chemical bonding. Detailed results are demonstrated on linear and cyclic C_*n*_ clusters (*n* = 3–10) trained on extensive B3LYP/aug-cc-pVTZ data. The described method facilitates PES learning by reducing the root mean squared error (RMSE) by a factor of 5 relative to the *s*-function formulation and by a factor of 20 relative to the conventional PIP approach. This is equivalent to using CDM and an *sp* basis with a PIP of order *M* to achieve the same RMSE as with the conventional method with a PIP of order *M* + 2. Implications for large-scale problems are discussed using the case of the PES of the C_20_ fullerene in full permutational symmetry.

## 1 Introduction

Computational studies of molecular spectra and chemical reactions require potential energy surfaces (PESs), often of high accuracy, that encompass key stationary structures, dissociation products and isomerization pathways, and possess all symmetry related properties originating from the presence of identical nuclei.^1^ The past few years have seen quite a remarkable progress in the learning of PESs as (global) analytic functions of the internal coordinates.^2−19^ PES representation in terms of easily calculable functions is particularly important because (1) in quantum vibrational approaches the evaluation of matrix elements over the potential operator requires substantial numbers of potential functions evaluations in many dimensions, and (2) in classical approaches the intermolecular forces, derived from the potential energy, determine the structure, reactivity, dynamics, response to external fields, and other properties. In this regard, learning the global PES of small and sometimes medium size molecules based on data generated at a reasonably high-quality electronic structure level is relatively straightforward, as demonstrated by many recent calculations.^2,10,20^ Notably, molecular systems that include a number of identical nuclei freely exchanging positions among themselves, as in neutral and protonated water clusters and other related systems, have received due attention as especially challenging.^21−23^

The importance of like-nuclei permutational invariance has been discussed at length previously,^7,8^ and in the present work, we use permutationally invariant polynomials (PIPs) for their straightforward implementation to enforce this symmetry. Of further importance is the fact that there are dramatically fewer PIP terms for a given polynomial degree than unsymmetrized monomials,^9^ which allows for many fewer data points needed for learning the model, e.g., for a cluster/molecule with ten identical nuclei a PIP of degree 4 contains 211876 primitive monomials contracted into 36 PIPs. On the other hand, applying the method of PIPs to progressively larger molecules and clusters quickly becomes computationally intractable due to the factorial scaling of the permutational space. We have previously tackled this problem by decomposing a PES into fragments and fitting each fragment with independent linear expansion coefficients, resulting in many fewer PIP terms needed for a high-quality PES fit.^24,25^ Here we describe further improvements that can be made and evaluate the model’s perspective robustness.

At this point, it is useful to introduce the basic premises of the proposed PES learning approach. The electronic energy in a post-HF theory is written as a sum of the HF energy in an AO basis and a correlation energy,
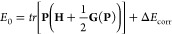
1where **P** is the bond order charge density matrix, **H** is the one-electron core Hamiltonian and **G**(**P**) is the density-dependent two-electron part of the Fock matrix.^26^ It is clear that the energy can be decomposed into a combination of unknown density elements *P*_*ab*_ (*a, b* being nuclear indices) weighted by corresponding and known Hamiltonian elements *H*_*ab*_ and augmented by an unknown correction term Δ*E*_corr_. The PES as a whole is a nonlocal function of **R** (from here on assumed a 3*N* vector of the atomic Cartesian coordinates), i.e., a change in any internal coordinate necessarily leads to a change in the PES. However, the density elements of **P** behave more locally in the same way that atomic charges and bond orders do. That is to say, a change in an internal coordinate describing the internuclear distance *R*_*ab*_ may have only a minor effect on the density element describing the electron population of atom *c* or the bond order of the pair of atoms *c-d*. We will demonstrate that this property is a major reason for significantly fewer PIPs needed for learning the PES via eq 1, even if one neglects the two-electron contribution **G**(**P**) in the so-called Core-Hamiltonian Model (CHM) approximation, as was demonstrated by us using an *s*-function treatment of the density.^24^ Incidentally, in the present work, we continue to use the CHM model albeit with some important modifications, which will be explained in detail below.

Second, the correlation energy term Δ*E*_corr_ is known to be a slowly varying function of the internal coordinates **R** and can perform the role of correction energy when using eq 1 to train the PES on data from a correlated level of theory, e.g., DFT, MBPT, CCSD(T), *etc*.^24^ This premise is based on the popular delta-machine-learning (Δ-ML) approach of constructing a PES in a two-stage process: (1) using an extensive set of inexpensively produced low-level data to sample the phase space of interest and train a low-level PES, followed by (2) generating a much smaller set of high-level “expensive” data to retrain the initial model to a high-level standard, referred to as Δ-ML.^27,28^ Here, we use the Δ-ML principle and build upon the aforementioned CHM approach^24^ to train a PES, however, we expand our original formulation of the electron density from *s* functions to *s* + *p* functions in order to determine the importance of the latter. We propose specifically to improve the training and significantly reduce the number of PIP terms needed to train the model. Obviously, the role of *p*-functions in the chemical bonding of the second and lower-row elements is crucial and should not be ignored in physically inspired models such as the present one. Generally speaking, it is desirable to train mathematical models using as low order a polynomial function as possible to minimize introducing nonphysical behavior into the model. Others have attested to this in similar efforts to train PESs using novel ML-based techniques.^6,29,30^

To evaluate our approach numerically and to further validate it on systems with moderate and high permutational symmetry with no constraints, we have chosen a series of charge-neutral carbon clusters C_*n*_ with *n* = 3–10 with strong chemical bonding where the bulk of interaction occurs via *sp*-hybridized valence orbitals resulting in both cumulenic and acetylenic forms of linear and monocyclic nuclear configurations.^31−45^ The C_20_ fullerene has also been considered as a relevant case study.

## 2 Theory and Computational Methods

Here, and as was demonstrated previously,^24^ we take the approach of constructing our model using the Hartree–Fock theory. To put it more specifically, the molecular PES is modeled as a sum of the one-electron Hamiltonian (the core Hamiltonian) contribution, a correction energy interpreted as a correlation energy, and the nuclear repulsion energy,
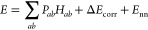
2with the **R** dependence assumed for all terms. As mentioned above, we neglect the two-electron contribution to the energy, i.e. the electron–electron repulsion and exchange interaction contained in **G**(**P**), due to its dependence on the density matrix specifically because our present goal is to use linear regression for PES learning. (A linearized approximation for the **PG**(**P**)/2 term in eq 1 is being evaluated independently by some of us.) Also, for clarity of method demonstration we at this stage assume a homonuclear molecule with a single Gaussian *s*-type orbital per atom-center *a* in the above notation and also assume all the associated permutational symmetry properties with this choice. Below we will generalize this notation to *sp*-type basis sets and will make necessary adjustments for permutational symmetry. The matrix elements *H*_*ab*_ (see below) are the one-electron Gaussian integrals, which are known analytically, as is the nuclear repulsion energy term. The unknown factors are the *P*_*ab*_ density matrix elements and the Δ*E*_corr_ energy, and they are to be learned using data derived from *ab initio* calculations.

We start with the correction (*a la* correlation) energy term which is a linear combination of PIPs used in the conventional fitting of PESs
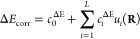
3where *c*_*i*_^Δ*E*^ are the linear coefficients, *u*_*i*_(**R**) is the *i*-th PIP of the power 0 < *m*_*i*_ ≤ *M* constructed in the usual way by summing over symmetrized monomial products *j*,
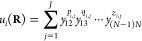
4

We use Morse variables *y*_*ab*_^*p*_*i*, *j*_^ = *exp* (− *p*_*i*, *j*_*d*_*ab*_/*t*_0_) where *p*_*i*, *j*_ is an integer power, *d*_*ab*_ = |**R**_*a*_ – **R**_*b*_| is the internuclear distance, and *t*_0_ is a range parameter. The integer powers satisfy the relation *p*_*i*, *j*_ + *q*_*i*, *j*_ + ··· + *z*_*i*, *j*_ = *m*_*i*_. All combinations of a given integer set, which satisfy the like-nucleus permutational symmetry rules, are considered for each *m*_*i*_. For a chosen maximal polynomial power *M* this determines the PIP basis size *L*.

The density matrix is divided into two parts: the one-center elements containing the permanent *s*-electron population with a correction term,
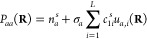
5aand the two-center elements
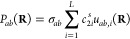
5bwith the shape functions defined respectively as
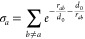
6a
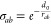
6bas was proposed in our preceding work.^24^eqs 5a and 5b, the coefficients *c*_1*i*_^*s*^ and *c*_2*i*_^*s*^ are one and two-center variational parameters. The newly introduced one-center and two-center functions are covariantly symmetrized conventional PIPs, namely,
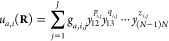
7a
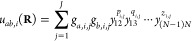
7b

They are similar to eq 4 but with a separate exponential range parameter *d*_0_. Based on the scheme, which is described in the Supporting Information (SI) Section S.1, with which the *g*_*a*, *i*, *j*_ phase factors are constructed, the one-center PIPs, and therefore the one-center *s*-density matrix elements *P*_*aa*_, are transformed into one another on an *a-b* permutation as *u*_*a*, *i*_ ↔ *u*_*b*, *i*_. The two-center PIPs and the corresponding *s*-density elements *P*_*ab*_ are also transformed into one another on a *b-c* permutation as *u*_*ab*, *i*_ ↔ *u*_*ac*, *i*_ but are invariant on an *a-b* permutation *u*_*ab*, *i*_ ↔ *u*_*ba*, *i*_ as is evident from eq 7b. The eqs 7a and 7b form the basis for subsequent derivation of the higher angular momentum expressions, and for convenience, they are referred to as formulas **F1** and **F2**, respectively.

We next note some important permutational symmetry properties of the density matrix with the inclusion of *p* orbitals, i.e. *x*, *y*, *z* functions. Consider, for the sake of demonstration, the density of an H_2_-like molecular fragment of a larger colinear cluster described with atom-centered *s* and *x* basis functions,
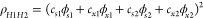
8awhere the expansion coefficients are *c*_*s*1_ ≠ *c*_*s*2_ and *c*_*x*1_ ≠ *c*_*x*2_. The 1–2 permutation also implies swapping (relabeling) the basis functions φ_*s*1_ → φ_*s*2_ and φ_*x*1_ → φ_*x*2_ which incidentally must be identical in order for the two nuclei to be considered identical. The density of the permuted configuration is similarly relabeled as (H2H1) and must be physically unchanged
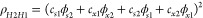
8b

**Scheme 1:**
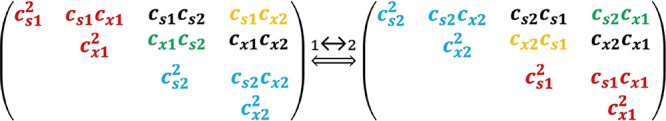
Upper Triangle of the Density Matrix is Represented in the (*s*_1_,*x*_1_,*s*_2_,*x*_2_) Basis for a Homonuclear Diatomic with the Nucleus Centers Labeled 1 and 2 with One *s* and One *x* Type Basis Function per Center. The density elements are expressed in terms of the basis function coefficients *c*_*s*1_, *c*_*x*1_, *c*_*s*2_, *c*_*x*2_. The effect of nucleus permutation, denoted as 1 ↔ 2, is demonstrated. The color scheme is black (unchanged density elements), red/blue/green/yellow (changed density elements)

Expanding the squares in both eqs 8a and 8b, and matching corresponding terms produces transformation properties of the full-density matrix block describing the two nuclei, presented in Scheme 1. The one-center elements transform into each other as *c*_*s*1_^2^ ↔ *c*_*s*2_^2^, *c*_*x*1_^2^ ↔ *c*_*x*2_^2^ and *c*_*s*1_*c*_*x*1_ ↔ *c*_*s*2_*c*_*x*2_ as the two nuclei are exchanged. The two-center elements of the same kind (*c*_*s*1_*c*_*s*2_ and *c*_*x*1_*c*_*x*2_) are obviously unchanged. The behavior of the above elements is consistent with the formulas **F1** and **F2** which state that the one-center elements and two-center-same-type elements transform onto each other, respectively.

On the other hand the products *c*_*s*2_*c*_*x*1_ and *c*_*x*2_*c*_*s*1_, being the two-center mixed-type elements, must be exchanged on the permutation if the density is to be unaltered (as is shown in Scheme 1 with the yellow/green combinations). However, this is inconsistent with **F2** which is designed for pure *s*-type density, and via the PIP symmetrization it erroneously leaves the aforementioned elements unchanged. To correct this, additional permutational symmetrization of the *c*_*s*2_*c*_*x*1_ and *c*_*x*2_*c*_*s*1_ density matrix elements is required. The following linear combination of the primitive two-center-mixed-type elements is proposed,
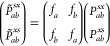
9where *a* and *b* are two identical nuclei and *f*_*a*_ is a function of the nuclear configuration. One choice for such a function is
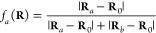
10where **R**_0_ is an origin, such as the center of the system’s geometry (used here), center of nuclear charge, or other similar choices, and which has the properties 0 ≤ *f*_*a*_(**R**) ≤ 1 and *f*_*a*_(**R**) + *f*_*b*_(**R**) = 1, ∀ **R**. It is readily seen that since on an *a-b* permutation we have *P*_*ab*_^*sx*^ = *P*_*ba*_^*sx*^ and *P*_*ab*_^*xs*^ = *P*_*ba*_^*xs*^, as guaranteed by **F2**, then the symmetrized elements transform as *P̃̃*_*ab*_^*sx*^ ↔ *P̃̃*_*ab*_^*xs*^, reflecting the correct physical behavior. For the full *sp* basis set, the same matrix equation is used for the other elements of this sort, i.e. *sy* and *sz*. And similarly, we write for the elements of the sort shown below,
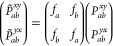
11

It is also evident that the Hamiltonian matrix elements transform in the same fashion as the corresponding density matrix elements upon like-nuclei permutations. More generally, a like-nucleus permutation is a unitary transformation of the Hamiltonian, **H** ′ = **UHU**^*T*^, and simultaneously of the density matrix, **P** ′ = **UPU**^*T*^, so that invariance of the electronic energy is preserved: *E*_0_ = *tr*[**P** ′ **H** ′ ] = *tr*[**PH**] owing to trace invariance under cyclic permutations.

Implementing an explicit *sp*-basis, we introduce a generic notation for the overlap and the core Hamiltonian,
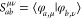
12a
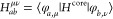
12bwhere φ_*a*, μ_ is a primitive GTO with the angular momentum μ component centered on nucleus *a*. Presently, we use the following definition: μ, ν = *s* represents the 1*s* primitive GTO, and μ, ν = *x*, *y*, *z* represents the 2*p* primitive GTO in the standard convention,
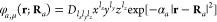
13where *D*_*l*_*x*_*l*_*y*_*l*_*z*__ is the normalization constant and *l*_*x*_, *l*_*y*_, *l*_*z*_ are the orbital quantum number integers, e.g. 000 for *s*, 100 for *p*_*x*_, etc. (Full expressions of the integrals in eqs 12 are provided in Section S.2.) Presently we do not distinguish between 1*s* and 2*s* electrons for reasons of numerical tractability and of only minor involvement of the 1*s* shell in chemical bonding. Thus, for a second-row atom, *s*-function implies a collective 1*s*2*s* “orbital”, and specifically for carbon, it implies a 4 *s*-electron and 2 *p*-electron virtual C atom.

Similarly, the density matrix in its generic closed-shell form is defined as , with *d*_*Ia*_^μ^ being the (real) coefficients of the basis function φ_*a*, μ_ in the doubly occupied MO *I*. These coefficients do not need to be known *a priori* since, in fact, the *P*_*ab*_^μν^ elements are represented using PIPs. Thus, to extend eqs 5*a* and 5*b* to s*p*-basis, we write for the one-center density elements,
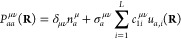
14aand for two-center density elements,
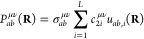
14bwhere the shape functions are modified from their *s*-basis counterparts as
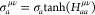
15a
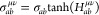
15b

This modification is a heuristic approach to enforce particular symmetry of the density matrix, which is evident in linear and planar configurations. For a planar molecule in the *xy*-plane all *sz*, *xz*, and *yz* density elements must be identically zero and be odd functions of *z*. By construction eqs 5*a* and 5*b*, if applied directly to the matrix elements involving *sp*-functions, do not possess this symmetry. And since the Hamiltonian naturally fulfills this requirement, the choice of an odd sigmoid-shaped function, commonly used in neural networks (NN),^2^ in eqs 15a and 15b imparts this symmetry to the density matrix. Our extensive exploratory calculations have shown a moderate, 10–20%, improvement in the RMSE with the functional implementation of eqs 15a and 15b versus the unsymmetrized formulation.

The total number of electrons at a given nuclear configuration is calculated in the usual trace-conservation fashion,
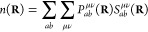
16with configuration-dependence arising due to the approximate form of the density matrix. This quantity is used in the fitting of the energy as a way to control the behavior of individual elements (see below). The total molecular charge is calculated as *Z*(**R**) = *n*_proton_ – *n*(**R**).

To implement the above formulation for practical applications, we must address the fact of invariance of the energy and the molecular charge with respect to spatial rotations. A rigid-body rotation is a unitary transformation of the nuclear position vector: **R** ′ = **UR**, where **U** is a direction cosine matrix. For a density described only by *s*-functions we have **H**(**R** ′) = **H**(**R**) and **P**(**R** ′) = **P**(**R**), i.e., both the Hamiltonian and the density matrices are invariant to rotations. However, inclusion of *p*-functions (*x*, *y*, *z*) makes **H**(**R**) and **P**(**R**) orientation-dependent. The Hamiltonian matrix has the rotational covariance built in by virtue of explicit dependence on Cartesian positions of atoms and does not require special treatment. On the other hand, the parameterized density matrix is not derived from the secular equation, as is usually the case in *ab initio* calculations, but is learned *a priori* by PIPs in a body-fixed (BF) frame and thus, by construction has no rotational covariance. Our approach to this problem is to construct a uniquely defined BF coordinate system, i.e. the direction cosine matrix **U**, and parametrize the density matrix in BF to make the energy orientation-independent. Details of this procedure are given in the Section S.3.

In summary, for a system consisting of a number of identical the second row atoms with a primitive *sp* basis (incorporating the core electrons), as will be presented below for various C_*n*_ clusters, the number of density matrix elements used in the learning of the PES is 26, i.e. with 10 unique elements originating from the atom terms (*ss, sx, sy, sz, xx, xy, xz, yy, yz, zz*) and 16 elements originating from atom–atom terms: (*s,x,y,z*)(*s,x,y,z*)^*T*^. Additional terms are derived from the correction energy Δ*E*_corr_, (cf. eq 3). Therefore, for a PIP basis size *L*, where by the present convention we count the constant term *c*_0_^Δ*E*^ separately, the total number of variational parameters entering eq 2 is *L*’ = 27*L* + 1.

## 3 Results and Discussion

In this section, we discuss the most important numerical results of applications to linear and cyclic C_6_, C_10_, and the C_20_ fullerene, while providing additional numerical data on these and other C_*n*_ (*n* = 3–5, 7–9) clusters in Sections S.4-S.11. For comparison, we examine three types of models of a PIP of degree *M*: the conventional model PIP[*M*] which is a direct fit of PIPs to the electronic energies, the Core Hamiltonian Model with only *s*-functions CHM_s[*M*], and the corresponding mode with *sp*-functions CHM_sp[*M*]. Prefacing the description of the numerical data, we mention that the fitting of eq 2 to a set of *ab initio* points is done in the following steps:

1. An NVE trajectory is propagated “directly” at the B3LYP/aug-cc-pVTZ(*spd*) level of theory for 10000 steps with 1 fs integration time and with the total energy equal to the harmonic ZPVE;
2. The resulting set is split into two subsets of 5000 points each: the odd-numbered points comprise the training set **TR** and the even-numbered points comprise the testing set **TEST**; for the low order PIPs overfitting is unlikely, but for control purposes we test the trained models on the **TEST** data set energies and incidentally on the corresponding gradient set named **TEST-G**;
3. A least-squares problem is formed using **TR** as follows: the energies are given a weight of 1.0 and the electron populations (eq 16) are given a weight of 0.001, resulting in a weighted 10000 data set of points learned with a PIP of degree *M* and with *L*+1, 3*L*+1 and 27*L*+1 total variables for the PIP[*M*], CHM_s[*M*] and CHM_sp[*M*] models, respectively;
4. To train the CHM_sp[*M*] models, a free carbon atom is assumed to exist in a spherically averaged {1s2s}^4^2p^2^ configuration. The permanent atomic populations entering eq 14a are set as follows: *n*_C_^*s*^ = 4, i.e., 1*s* and 2*s* orbitals comprise a single shell, and *n*_C_^*x*^ = *n*_C_^*y*^ = *n*_C_^*z*^ = 2/3. We have also considered the {1s2s}^3^2p^3^ configuration by setting *n*_C_^*s*^ = 3, and *n*_C_^*x*^ = *n*_C_^*y*^ = *n*_C_^*z*^ = 1, although no tangible improvement in the RMSE was observed;
5. For each value of the nonlinear parameters (*d*_0_, *t*_0_, α) the collective coefficients **c** are found by a standard linear regression using a QR decomposition^46^ as implemented in the DGELS subroutine supplied within the MKL software suite. Additional optimization of the nonlinear parameters is performed on a uniform grid to find the smallest RMSE, and thus all the results reported below correspond to variationally best sets of these parameters.

### 3.1 Calculations of C_6_ in Cyclic (^1^A_g_ @ D_*3h*_) and Linear (^3^Σ^–^_g_) Forms

It has been noted that C_6_ exists in two low energy forms, a distorted hexagonal (D_*3h*_ ring) structure in a singlet electronic state and a triplet electronic state (*D*_∞h_ linear). Both these structures have the cumulenic type of bonding, i.e., nearly identical double bonds. High-level electronic structure calculations, CCSD(T)/cc-pVTZ, show that the cyclic form is the more stable one by ∼13 kcal/mol.^43^ However, the density functional theory, as reported here at the level of B3LYP/aug-cc-pVTZ(*spd*), identifies the linear structure as the lowest in energy by ∼6 kcal/mol. A recent study at the PBE/Def2-TZVPP level of theory, along with infrared spectroscopy measurements^36^ found a linear C_6_ in the triplet state to be more stable than the cyclic C_6_ in the singlet state by ∼0.26 eV. Since experimental measurements identify both forms as viable,^31,36^ we also consider the cyclic and linear structures in the present calculations. The linear C_6_, with its triplet configuration character, is moreover interesting due to the application of the present formulation that was derived for a closed shell molecule, and whether any serious procedural issues arise as a result.

**Figure 1:**
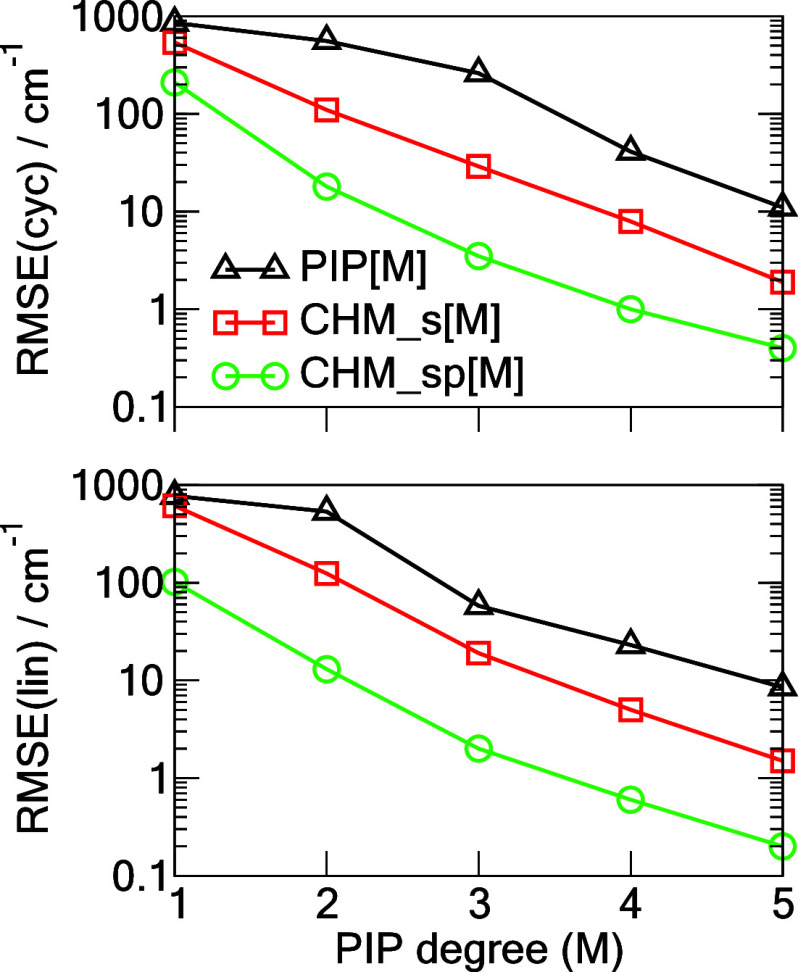
RMSEs of three models, (PIP[*M*], CHM_s[*M*], CHM_sp[*M*]) trained on **TR** and tested on **TEST** for the cyclic singlet state (cyc) and linear triplet state (lin) C_6_ as functions of the PIP degree *M*. Line fits to the log_10_(RMSE) data yield the following slopes for the cyclic C_6_: −0.491, −0.605, −0.670; and respectively for the linear C_6_: −0.528, −0.661, −0.675.

We find it imperative to examine PES learning using PIP degrees starting at the lowest level M = 1 (where the number of nonconstant PIPs is 1 for all C_*n*_ clusters) and proceeding by increments of 1 to the maximally allowable degree as controlled in practice by the data set size, permutational symmetry, and other numerical considerations. Our goal is to demonstrate whether the smallest possible sets of low-order PIPs can lead to well-learned PESs. Similar strategies have been reported in recent years in various NN-PIP formulations.^4,11,18,19,29^Figure 1 shows a summary of these tests of the cyclic and linear C_6_ for the three models considered. Convergence of the RMSE with PIP degree *M* is approximately exponential for the CHM_s and CHM_sp models but appears to be much slower for the conventional PIP approach especially at the small values of *M*. One can observe a major improvement in the RMSE of CHM_sp versus PIP for a given *M* which amounts to CHM_sp[*M*] performing comparably to PIP[*M*+3]. It is expected that at M = 5 the CHM_sp model is essentially converged, with the corresponding RMSEs for the cyclic and linear C_6_ equal 0.4 and 0.2 cm^–1^, as detailed in Table 1. A similar high quality of the fit is observed also for the total molecular charge or, equivalently, the total number of electrons. In the cyclic C_6_, the total charge is conserved within one part in a few thousand despite the weight of the charge data being 0.001 (1.0 for the energy). A worse conservation is noted for the linear C_6_ in the CHM_sp[5] model with the electron population RMSE of 0.11 of the total 36 electrons.

**Table 1:** C_6_ Training and Testing RMSE Data, in cm^–1^, for the Cyclic Singlet (cyc) and the Linear Triplet (lin) at the B3LYP/aug-cc-pVTZ(*spd*) Level of Theory^a^

**model**	*L*	*L*’	*d*_0_	*t*_0_	α	**TR**	**TEST**	**TEST-G**
PIP[5](cyc)	85	86	4.6			11	11	398
CHM_s[5](cyc)	85	256	2.5	2.0	1.2	1.9	1.9 {7.7e-4}	91
CHM_sp[5](cyc)	85	2296	2.0	2.5	1.5	0.4	0.4 {1.2e-4}	36
PIP[5](lin)	85	86	4.4			8.5	8.5	439
CHM_s[5](lin)	85	256	5.0	2.5	0.1	1.5	1.5 {6.5e-4}	97
CHM_sp[5](lin)	85	2296	2.0	3.0	0.1	0.2	0.2 {0.11}	37

a For the three reported models: PIP[*M*], CHM_s[*M*] and CHM_sp[*M*], the PIP degree is M = 5, and the corresponding number of PIP terms (*L*) and the total number of variational parameters (*L*’) are provided. The training and testing sets **TR** and **TEST** each contain 5000 points obtained from an NVE trajectory propagated at the harmonic ZPVE levels of 6006 for the cyclic and 5121 cm^–1^ for the linear isomer, respectively. **TEST-G** contains gradient RMSE data, in cm^–1^/bohr, at the points of **TEST**. The optimized nonlinear parameters *d*_0_, *t*_0_, α are reported in a.u. The numbers in {} for **TEST** are the RMSEs of the total molecular charge

**Figure 2:**
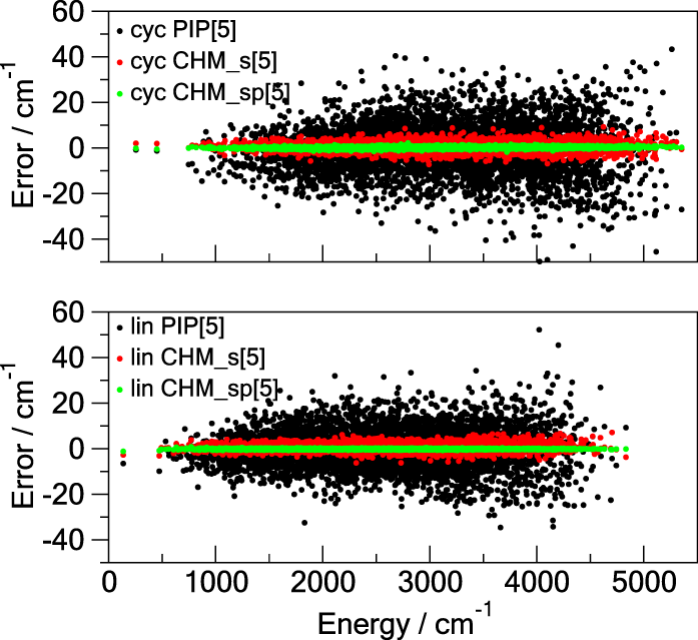
Errors of the **TR**-trained models on the **TEST** data as functions of the potential energy above the minimum for the cyclic (cyc) and linear (lin) C_6_. All three models use the PIP of total degree 5: the conventional PIP[5] (black dots), the *s*-density CHM_s[5] (red dots), and the *sp*-density CHM_sp[5] (green dots).

A note of warning is needed for the possibility of overfitting, especially considering that the CHM_sp[*M*] model contains 27 times the number of variational parameters compared to the corresponding PIP[*M*] one. The calculations show, however, that for the CHM_sp[*M*] model both the training and testing RMSEs are essentially the same (cf. Table 1). Furthermore, the RMSE of the test set gradient is about an order of magnitude smaller for CHM_sp[*M*] than for PIP[*M*]. Figure 2 provides an additional descriptive comparison where the test set errors are shown as functions of the potential energy. Of particular interest is the ability of CHM_sp[*M*], and to a lesser degree of CHM_s[*M*], to have the error distributed approximately uniformly with the energy of the configuration, unlike that of PIP[*M*] which shows severe degradation of the fit away from the global minimum (the zero of the energy). This imparts certain confidence to reliability of the CHM_sp[*M*] model.

**Figure 3:**
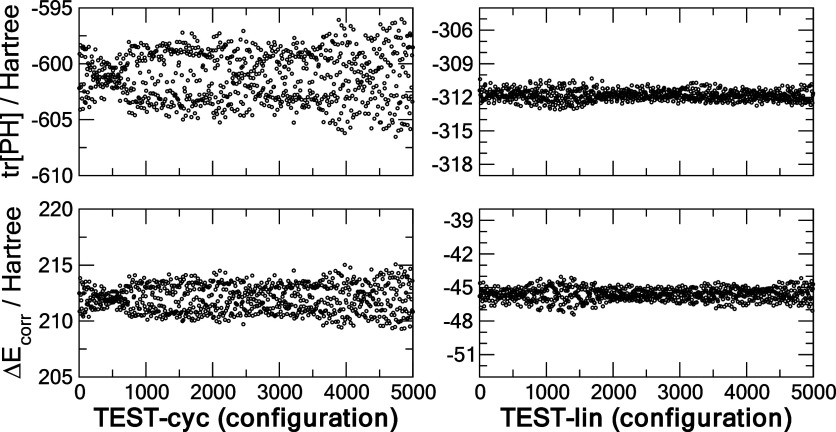
Energy decomposition of the cyclic and linear C_6_ using the CHM_sp[5] models trained on **TR** and showing the **TEST** data of the two principal components: the core Hamiltonian energy tr[**PH**] and the correction term Δ*E*_corr_.

A deeper insight into the properties of the trained CHM_sp[*M*] model can be gained by the decomposition of the electronic energy of eq 2 into the density matrix component and the correction term. In Figure 3 we show the tr[**PH**] and Δ*E*_corr_ components for the cyclic and linear C_6_ from the corresponding test sets. Not surprisingly, the magnitude of the correction is smaller than that of the core energy in both isomers, yet the corresponding fraction |Δ*E*_corr_/tr[**PH**]| is substantially greater in the cyclic than in the linear C_6_. This is likely the result of a greater electron–nucleus attraction energy in the more compact ring (compare the average of −600 and −312 hartree) and by a subsequent overcompensation in the fitted Δ*E*_corr_ energy in the ring structure compared to the linear one.

**Figure 4:**
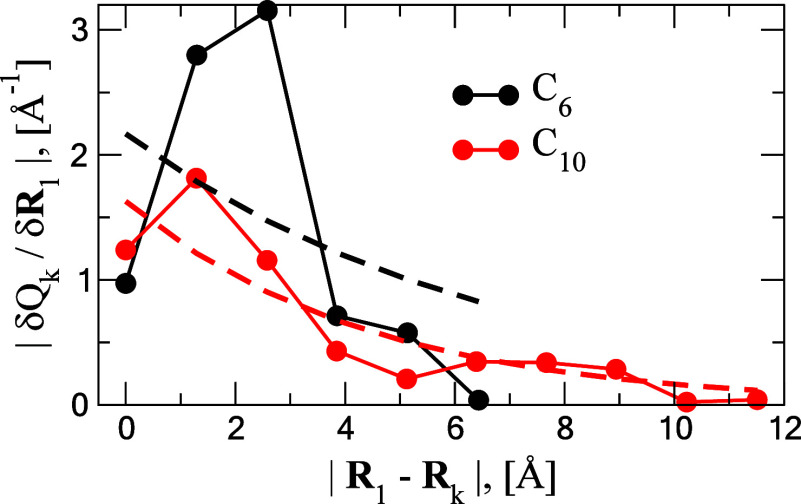
Derivatives of the B3LYP/aug-cc-pVTZ(*spd*) Mulliken populations *Q*_*k*_ on atoms *k* = 1, ..., *n* taken with respect to the Cartesian coordinate **R**_1_ for the two C_*n*_ linear chains with *n* = 6 (black data points and curves) and *n* = 10 (red data points and curves). The dashed lines are exponential fits of the form *A* exp (− |**R**_1_ – **R**_*k*_|/*B*) with *B* = 6.668 and 4.375 Å for C_6_ and C_10_, respectively.

To test the premise of locality of the density matrix elements in the CHM model, exposed in the Introduction, we consider a simple test on the linear C_*n*_ chains examined here, where the definition of locality is more straightforward to illustrate than for the cyclic C_*n*_ forms. In a chain C^1^=C^2^=···=C^n-1^=C^n^ we take as an observable the derivative of the gross Mulliken population *Q*_*k*_ of one of the terminal atoms, chosen here as C^1^, with respect to the Cartesians of all the other atoms (including C^1^), i.e., the quantity  for *k* = 1 - *n*, and plot it as a function of the distance, |**R**_1_ – **R**_*k*_|. Figure 4 shows these plots for the linear C_6_ and C_10_ triplets, plotted together to better demonstrate the trends. One can observe large derivative norms of the atomic populations for the atoms close to the terminal carbon (*k* = 1, 2, 3) and progressively smaller norms for *k* > 3. This is especially evident in the larger C_10_ where the distance from the terminal carbon extends to ∼12 Å. Fitting the data to a single exponential form, *y* = *A* exp (− *x*/*B*), produces the range of decay parameters *B* = 6.7, 3.9, 6.6, 7.2, 4.4 Å for C_*n*_ (*n* = 6–10), respectively, with the mean of ∼5.7 Å and a standard deviation of ∼1.5 Å. Based on these data of atomic charge derivatives we expect an exponentially decaying distance dependence of the local density elements *P*_*aa*_ and *P*_*ab*_ (for an adjacent *a-b* nuclear pair) on the coordinates of the other atoms in a cluster, which greatly facilitates PES learning via use of eq 2.

It is insightful to estimate the contribution of the *d*-functions to the atomic charges and, therefore to the density matrix, especially considering that the CHM models are limited to *sp*-functions. Our calculations using B3LYP/aug-cc-pVTZ(*spd*) of several stationary points of the linear triplet and cyclic singlet show that the contribution of the *d*-functions in Mulliken atomic populations in C_6_ and C_10_ is in the 1.5–2.3% and 2.5–3.3% ranges, respectively. These fractional contributions are comparable to the relative RMSEs of the fitted PESs using the CHM_sp[*M*] model with M = 1 and 2 (see Section S.12), suggesting the addition of *d*-functions into the CHM model will improve its performance for low order PIP bases.

### 3.2 Calculations of C_10_ in Cyclic (^1^A_g_ @ *D*_*5h*_) and Linear (^3^Σ^–^_g_) Forms

The provenance of C_10_ computational research dates back to the Hückel theory calculations of Hoffmann,^47^ which were followed by extensive *ab initio* studies confirming C_10_ to be a cyclic D_5h_ cumulene, a pentagon-like structure, with a regular decagon D_10h_ being a transition state connecting two D_5h_ pentagons.^44,45,48,49^ The linear C_10_, a triplet electronic state, is known from other computational studies to be substantially higher in energy than its cyclic C_10_ singlet state counterpart,^33^ and is presently found at the B3LYP/aug-cc-pVTZ(*spd*) to be 18147 cm^–1^ above the cyclic C_10_ singlet state global minimum.

**Figure 5:**
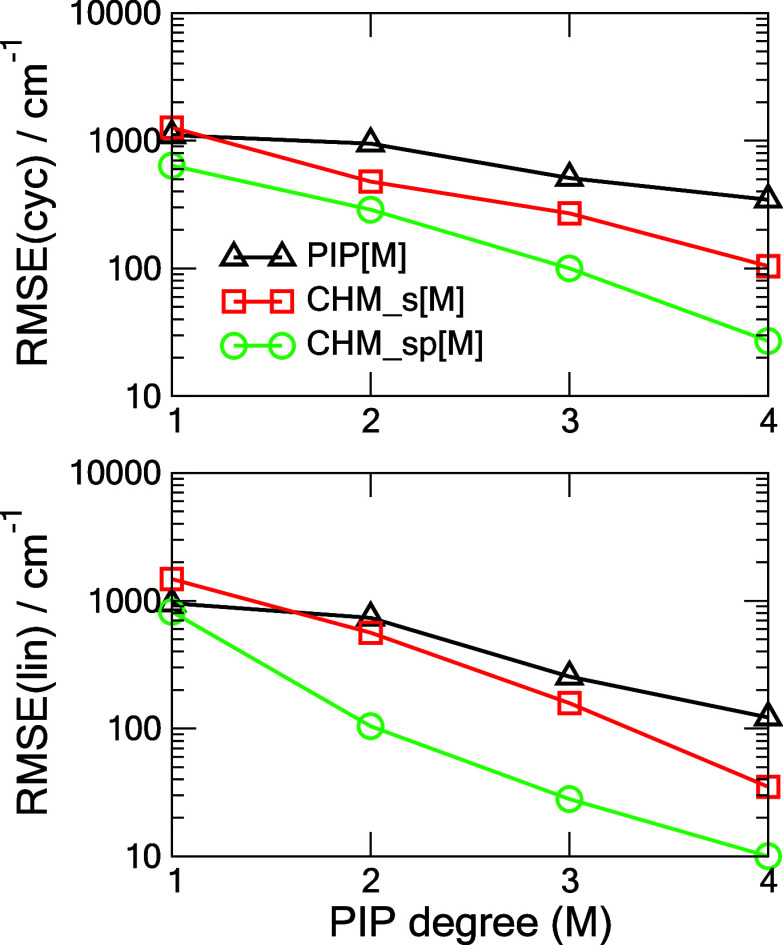
Test RMSEs of three models, (PIP[*M*], CHM_s[*M*], CHM_sp[*M*]) trained on **TR** and tested on **TEST** for the cyclic (cyc) and linear (lin) C_10_ as functions of the PIP degree *M*. Line fits to the log_10_(RMSE) data yield the following slopes for the cyclic C_10_: −0.178, −0.350, −0.457; and for the linear C_10_: −0.314, −0.543, −0.631, respectively.

**Figure 6:**
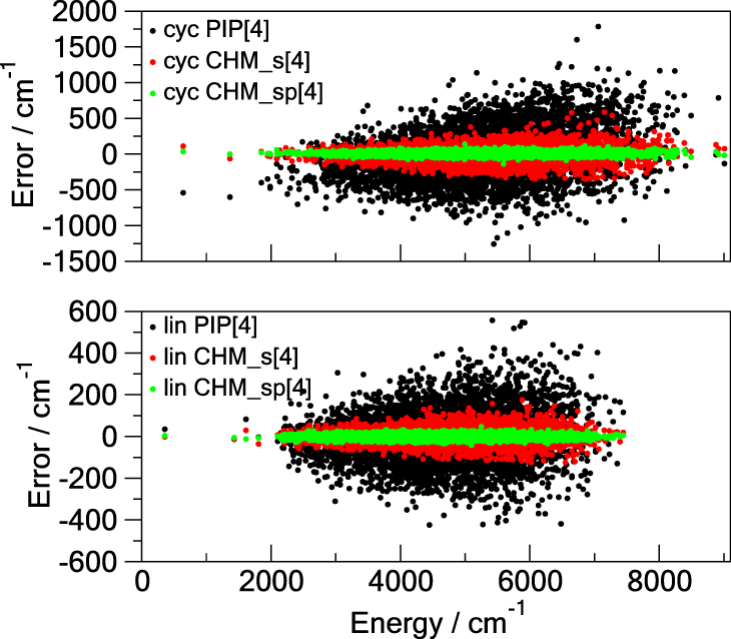
Errors of the **TR**-trained models on the **TEST** data as functions of the potential energy above the minimum for the cyclic (cyc) and linear (lin) C_10_. All three models use the PIP of total degree 4: the conventional PIP[4] (black dots), the *s*-density CHM_s[4] (red dots), and the *sp*-density CHM_sp[4] (green dots).

Following our procedure for C_6_, we train the models for both the ring and linear C_10_ using the B3LYP/aug-cc-pVTZ(*spd*) trajectory data and PIP degrees *M* ≤ 4. In Figure 5, we summarize the RMSEs for the test sets as functions of *M*, and in Figure 6 and Table 2, additional details of the fits for the specific case of M = 4 are provided. As is the case of C_6_, the RMSEs for the CHM_sp[*M*] model fall off approximately exponentially (i.e., nearly linearly on the log scale (as seen in Figure 5 caption) with *M*, both for the cyclic and the linear structure, but somewhat slower than in the respective C_6_ cases. Linear regressions of the RMSE data vs the PIP degree M reflect this trend. The CHM_sp[M] decays in the exponential regime twice as fast as the conventional PIP[M] model. Further, in C_6_ we found that RMSE{CHM_sp[*M*]} ≈ RMSE{PIP[*M*+3]}, for C_10_ the correspondence is closer to RMSE{CHM_sp[*M*]} ≈ RMSE{PIP[*M*+2]}. We note that a slightly better performance is observed for the linear conformers, in both C_6_ and C_10_, presently explained by potential energy sampling due to the lower ZPVEs and the corresponding NVE ensemble potential energies of the linear structures.

**Table 2:** C_10_ Training and Testing RMSE Data, in cm^–1^, for the Cyclic Singlet (cyc) and the Linear Triplet (lin) at the B3LYP/cc-pVTZ(*spd*) Level of Theory^a^

	*L*	*L*’	*d*_0_	*t*_0_	α	**TR**	**TEST**	**TEST-G**
PIP(4)(cyc)	35	36	1.0			345	345	8218
CHM_s(4)(cyc)	35	106	2.5	2.0	0.3	104	104 {0.02}	2658
CHM_sp(4)(cyc)	35	946	3.5	2.0	0.1	26	27 {9.5e-3}	1575
PIP(4)(lin)	35	36	1.6			122	122	4482
CHM_s(4)(lin)	35	106	2.0	6.5	0.1	35	35 {8.4e-3}	1091
CHM_sp(4)(lin)	35	946	5.0	2.0	0.1	10	10 {0.04}	524

a For the three reported models: PIP, CHM_s and CHM_sp, the PIP degree is M = 4, and the corresponding number of PIP terms (*L*) and the total number of variational parameters (*L*’) are provided. The training and testing sets **TR** and **TEST** each contain 5000 points obtained from an NVE trajectory propagated at the harmonic ZPVE levels of 10499 for the cyclic and 9395 cm^–1^ for the linear isomer, respectively. **TEST-G** contains gradient RMSE data, in cm^–1^/bohr, at the points of **TEST**. The optimized nonlinear parameters *d*_0_, *t*_0_, α are reported in a.u. The numbers in {} are the RMSEs of the total molecular charge

**Figure 7:**
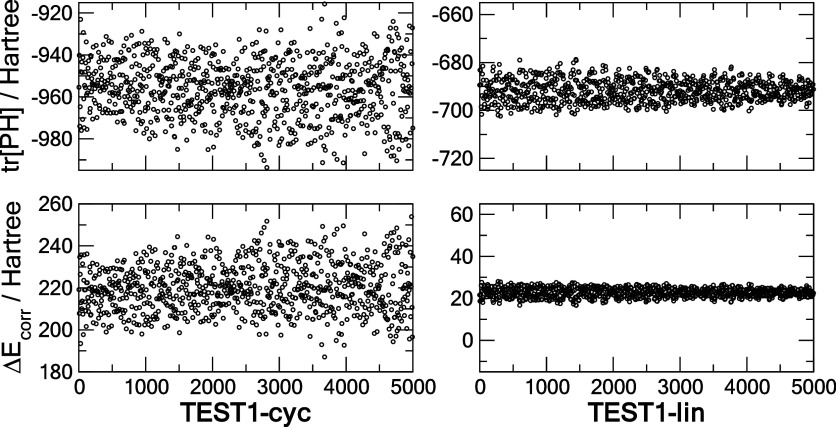
Decomposition of the electronic energy of the cyclic (cyc) and linear (lin) C_10_ using the CHM_sp[4] model trained on **TR** and showing the **TEST** data of the two principal components: the core Hamiltonian energy tr[**PH**] and the correction term Δ*E*_corr_.

The distribution of the error with the energy above the global minimum is shown in Figure 6. Notably, the CHM_sp[4] error stays more or less contained within a narrow strip on the [0, 9000] cm^–1^ range, unlike PIP[4] which tends to be smaller at the lower energies and expands toward the higher ones, as is also observed for C_6_. This is an important result that shows that CHM_sp is trainable to have both (*i*) a much smaller RMSE (as an average property) than the conventional PIP approach and (*ii*) a well balanced error distribution over the trained/tested range of energies. Briefly visiting energy decomposition, exposed in Figure 7, we observe a similar trend that the correction energy Δ*E*_corr_ is substantially smaller than the tr[**PH**] energy, and that the |Δ*E*_corr_/ tr[**PH**]| ratio is much smaller, by about an order of magnitude, in the linear as compared to the cyclic C_10_ structure.

As an interesting aside, we note that in the course of sampling the triplet surface, we found a low-energy cyclic configuration that appears to have been identified as viable only very recently using infrared spectroscopy and characterized at the PBE/Def2TZVPP level of theory.^36^ Our calculations with B3LYP/aug-cc-pVTZ(*spd*) place this cyclic triplet structure only 1737 cm^–1^ below its linear triplet counterpart, but with an activation barrier to linearity of 22737 cm^–1^, therefore confirming the respective stabilities of the two triplet state conformers. Additional details of the singlet and triplet surface topologies with higher levels of theory calculations and the ring-opening reaction profiles are shown in Section S.12.

### 3.3 Calculations of the C_20_ Fullerene

Being the smallest known fullerene, C_20_ exhibits sp^2^-hybridized bonding with expected much stronger many-body effects in the interaction potential than the sp-hybridized C_*n*_ clusters considered so far. The permutational symmetry space is 20! (∼ 2.4 * 10^18^ which makes generation of high order PIPs computationally prohibitive. Thus, our aim is to examine whether CHM_sp[*M*] can perform reasonably well for this system with a low order PIP. Just as for the monocyclic and linear C_*n*_ clusters, we generated a training and a test set from a trajectory propagated for 10000 steps but with a computationally less demanding level of theory (the pure functional) M06L with a nonpolarized valence basis 6-31G, and a lower total energy of ZPVE/16 = 1579 cm^–1^ corresponding to temperature ∼42 K. These data were used to train the three models for M = 1–3, 4* where the last term marked with an asterisk is an incomplete PIP. That is, we removed the 6 largest PIPs, by the number of monomials, from the complete M = 4 basis to make the calculations more manageable. The effect of this basis reduction on the RMSE was found to be negligible, as can be inferred from the data in Section S.13. Our observation is supported by recent research on machine learning-based PIP selection for PES fitting where individual PIPs are selected based on their contribution to lowering the RMSE rather than on their hierarchical order (Table 3).^50,51^

**Table 3:** C_20_ Training and Testing RMSE data, in cm^–1^, at the M06*L*/6-31G Level of Theory^a^

	*L*	*L*’	*d*_0_	*t*_0_	α	**TR**	**TEST**	**TEST-G**
PIP[4*]	29	30	1.2			68	68	6557
CHM_s[4*]	29	88	3.0	2.0	0.6	35	35 {0.02}	3263
CHM_sp[4*]	29	784	2.0	6.0	1.3	15	15 {3.7e-3}	1834

a For the three reported models, PIP, CHM_s and CHM_sp, the PIP degree is an incomplete M = 4*, and the corresponding number of PIP terms (*L*) and the total number of variational parameters (*L*’) are provided. The training and testing sets **TR** and **TEST** each contain 5000 points obtained from an NVE trajectory propagated at the harmonic ZPVE/16 level of 1579 cm^–1^, respectively. **TEST-G** contains gradient RMSE data, in cm^–1^/bohr, at the points of **TEST**. The optimized nonlinear parameters *d*_0_, *t*_0_, α are reported in a.u. units. The numbers in parentheses are the RMSEs of the total molecular charge

**Figure 8:**
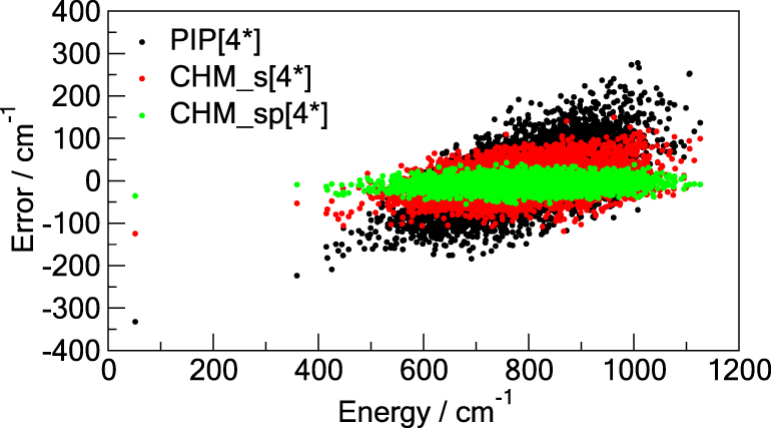
Errors of the **TR**-trained models on the **TEST** data as functions of the potential energy above the minimum for the C_20_ fullerene. All three models use the PIP of incomplete degree 4: the conventional PIP[4*] (black dots), the s-density CHM_s[4*] (red dots), and the *sp*-density CHM_sp[4*] (green dots).

Full results are summarized in Section S.13, while the most important results are presented below. A comparison of the RMSEs suggests a modest factor of 4 improvement for CHM_sp[4*] compared to PIP[4*] but is somewhat misleading. For instance, examination of the error as a function of the energy yields a more striking comparison, which is presented in Figure 8. The PIP[4*] model appears to not be able to represent the test data well missing the low energy region by more than 300 cm^–1^ as well as the high energy region by similar amounts. On the other hand, the CHM_sp[4*] data displays a much narrower and more uniform error distribution, suggesting a significantly better tempered model. The latter point is demonstrated on the example of normal mode cuts of the potential energy, shown in Section S.13. The CHM_sp[4*] model trained on **TR** exhibits remarkable fidelity for all the normal mode configurations and far exceeds the training set energies. At the lower energy range <400 cm^–1^, where few points were sampled we can instead examine the vibrational frequencies, summarized in Section S.13. One can see the CHM_sp[4*] model correlates closely with the DFT frequency data, with a linear regression of ω_CHM_sp_ = 1.023 * ω_M06L_ - 45 cm^–1^, i.e., recovering the true frequencies to within approximately 2%.

## 4 Conclusions

We describe a low-order PIP approach to fitting multidimensional PESs of systems with high permutational symmetry that is based on the Hartree–Fock expression for the energy with an added low-order correction term acting as the correlation energy. This approach is in the spirit of a divide-and-conquer strategy where smaller parts constituting the total can be handled much easier than the total. It was first introduced by us for learning the PESs of H_2_, CH_5_^+^ and H_3_CNO with very promising results,^24^ and more recently for calculating the cyclic singlet state C_10_ vibrational wave function using diffusion Monte Carlo.^25^ In the present work, we discuss the extension of the *s*-electron density to an *sp*-electron density and test it extensively on a series of carbon clusters C_*n*_ with *n* = 3–10 and as a separate exercise, the C_20_ fullerene. We show that the addition of *p*-electron density elements significantly improves the RMSE for these systems, which is an expected result for sp-hybridization chemical bonding. Specifically, our key findings include:

i. the density elements tend to behave as functions of the local environment unlike the PES itself, which is a function of the entire set of internuclear distances;
ii. the one-electron Hamiltonian contains 2-body and 3-body interactions via the integrals involving *s* and *p* basis functions, respectively, thus reducing the overall PIP order;
iii. the free-standing correction term Δ*E*_corr_ plays an important role in minimizing the RMSE;
iv. the combination of the above properties results in the core Hamiltonian model CHM_sp[*M*] performing roughly as the conventional model PIP[*M*+2] for a given PIP of degree *M*;
v. the CPU timings recorded for C_6_ yield the ratios of CHM_s[3]/PIP[3] = 8.3, CHM_sp[3]/PIP[3] = 12.9, CHM_s[4]/PIP[4] = 11.7, CHM_sp[4]/PIP[4] = 16.7, CHM_s[5]/PIP[5] = 13.9, CHM_sp[5]/PIP[5] = 14.0, i,.e. exposing roughly an order of magnitude increase in the computational effort for the CHM models. More importantly, however, the CPU timing ratio of CHM_sp[3]/PIP[5] = 0.96 shows that for the comparable RMSEs (∼4 and ∼11 cm^–1^, respectively) the low order CHM_sp model scales computationally comparably with the conventional high order PIP approach;
vi. at present, we have not observed any major differences in the learning of singlet vs triplet PES with the “spinless” CHM_s and CHM_sp models. In perspective, the inclusion of the two-electron Coulomb and exchange interactions, the **PG**(**P**)/2 term in eq 1, is intriguing from the electronic structure standpoint as one can differentiate between α and β electrons in PES learning of open-shell systems.

Going forward, we point out the need to address analytic gradients in the CHM_sp model, which is obviously important in MD simulations and surface explorations, and to examine alternative choices of the body fixed coordinate system used in learning the density matrix to avoid nonuniqueness at rare instances of high symmetry configurations. These points are presently being investigated by some of us.

## Supporting Information Available

Expressions for Gaussian matrix elements, symmetrization scheme for generating covariant polynomials, definition of a body-fixed coordinate system, C_*n*_ (*n* = 3–10, 20) training and testing data, and electronic structure and configuration data for C_6_ and C_10_. (PDF)
