# Supplementary material for: A Low-Order Permutationally Invariant Polynomial Approach to Learning Potential Energy Surfaces Using the Bond-Order Charge-Density Matrix: Application to Cn Clusters for n = 3–10, 20
Source: J Phys Chem A. 2024 Aug 29;128(36):7703–13. doi: 10.1021/acs.jpca.4c04281 (PMC11407436; doi:10.1021/acs.jpca.4c04281)
Supplement: Supplementary file 1 — jp4c04281_si_001.pdf [file jp4c04281_si_001.pdf]

Supporting Information for:

A Low Order Permutationally Invariant Polynomial Approach to Learning Potential Energy Surfaces Using the Bond-Order Charge-Density Matrix: Application to C_n Clusters for n = 3 – 10, 20

Jose Gutierrez-Cardenas,[1] Benjamin D. Gibbas,[1] Kyle Whitaker,[1] Martina Kaledin,[1,*] Alexey L. Kaledin[2,*]

[1] *Department of Chemistry & Biochemistry, Kennesaw State University, 370 Paulding Ave NW, Box # 1203, Kennesaw, Georgia, 30144*

[2] *Cherry L. Emerson Center for Scientific Computation and Department of Chemistry, Emory University, 1515 Dickey Drive, Atlanta, Georgia, 30322*

Table of contents

S.1 A symmetrization scheme for generating covariant polynomials....................................S2

S.2 Expressions for the one-electron (core) Hamiltonian matrix elements............................S3

S.3 Definition of a body-fixed coordinate system for density matrix parameterization.......S5

S.4 C_3 training and testing data..S7

S.5 C_4 training and testing data..S9

S.6 C_5 training and testing data..S10

S.7 C_6 training and testing data..S11

S.8 C_7 training and testing data..S13

S.9 C_8 training and testing data..S14

S.10 C_9 training and testing data..S15

S.11 C_{10} training and testing

data..S16

S.12 Electronic structure and configuration data for C_6 and C_{10} S18

S.13 C_{20} training and testing data..S24

S.1 A symmetrization scheme for generating covariant polynomials

Equations 6 take as input symmetrization phase factors $g_{a,i,j} = \pm 1$ that use nucleus indices 1 ... *a* ... *b* ... *N* to choose the phase for each monomial *j* such that a permutation of a pair of identical nuclei also permutes the two PIPs, which are labeled by *i*. In other words, PIP *i* describing nucleus *a* will transform into the corresponding PIP *i* describing an identical nucleus *b* upon the *a*-*b* permutation. This guarantees that the PES described by Eq. 1 is invariant under the said permutation and by extension under any permutation. This has been tested numerically on the systems considered in the present work.

The presently implemented scheme for choosing the symmetrizing phases is given below:

I. determine the number of non-zero powers in a monomial: M_NZ{monomial}

Ex: M_NZ$\{y_{12}^2 y_{13}^1 y_{14}^0 y_{15}^0 y_{23}^1 y_{24}^0 y_{25}^3\}$ = 4

II. define quantity MAX_SYM = min[N_ATOM - 1, M_NZ]

where N_ATOM is the number of atoms

III. define quantity MIN_SYM = M_NZ - MAX_SYM

IV. compute the number of times (F_k) the atom index (k) shows up in the monomial

Ex: for $y_{12}^2 y_{13}^1 y_{14}^0 y_{15}^0 y_{23}^1 y_{24}^0 y_{25}^3$

F_1=2, F_2=3, F_3=2, F_4=0, F_5=1

V. Compare F_k against the quantity F^* = max[MAX_SYM - 1, 1]:

a) set by default $b_k = 1$

b) if $F_k >$ MIN_SYM; then

if $F_k \geq F^*$; then $b_k = -1$

S.2 Expressions for the one-electron (core) Hamiltonian matrix elements

This section provides analytic expressions for the Gaussian integrals seen in Eqs. 12 of the main text. Here the integrals involving *s* functions will be given explicitly, with the corresponding *p* function integrals calculated as derivatives of the former. The normalized *s* function on center *a* with coordinate $\mathbf{R}_a$ is

$$\phi_a(\mathbf{r}) = \left(\frac{2\alpha_a}{\pi}\right)^{\frac{3}{4}} exp\left[-\alpha_a|\mathbf{r}-\mathbf{R}_a|^2\right] \qquad (S1)$$

where $\mathbf{r}$ is electron's coordinate. The Hamiltonian matrix element used in Eq. 2 of main text is

$$H_{ab} = T_{ab} + \sum_c V_{abc} \qquad (S2)$$

where the kinetic energy integral is

$$T_{ab} = \alpha_{ab}\left(3 - 2\alpha_{ab}R_{ab}^2\right)S_{ab} \qquad (S3)$$

and the overlap integral is

$$S_{ab} = \left(\frac{4\alpha_{ab}}{\alpha_a + \alpha_b}\right)^{\frac{3}{4}} exp\left[-\alpha_{ab}R_{ab}^2\right] \qquad (S4)$$

with $\alpha_{ab} \equiv \alpha_a\alpha_b/(\alpha_a+\alpha_b)$. The distance between two centers *a* and *b* is $R_{ab} = |\mathbf{R}_a - \mathbf{R}_b|$. The one-electron Coulomb integral involving two basis functions centered at *a* and *b* nuclear centers coupled over the nuclear center *c* with charge Z_c is

$$V_{abc} = S_{ab}Z_c\frac{erf[\sqrt{\alpha_a + \alpha_b}R_{1c}]}{R_{1c}} \qquad (S5.1)$$

where the *ab* centroid position vector is $\mathbf{R}_1 = (\mathbf{R}_a\alpha_a + \mathbf{R}_b\alpha_b)/(\alpha_a + \alpha_b)$ and $R_{1c} = |\mathbf{R}_1 - \mathbf{R}_c|$. For *a* = *b*, the above integrals reduce to

$$V_{aac} = Z_c \frac{erf\left[\sqrt{2\alpha_a} R_{ac}\right]}{R_{ac}} \qquad (S5.2)$$

$$T_{aa} = \frac{3}{2}\alpha_a \qquad (S5.3)$$

and for *a* = *b* = *c* the Coulomb integral reduces to

$$V_{aaa} = 2Z_a\left(\frac{2\alpha_a}{\pi}\right)^{\frac{1}{2}} \qquad (S5.4)$$

Matrix elements involving Cartesian *p* functions are derived by differentiation of the above integrals with respect to basis function centers, i.e. nuclear position $\mathbf{R}_a = \{X_a, Y_a, Z_a\}$, for the two and three center integrals:

$$T_{(ax)b} = \frac{1}{\sqrt{\alpha_a}}\frac{\partial}{\partial X_a}T_{ab} = -2\frac{\alpha_{ab}^2}{\sqrt{\alpha_a}}S_{ab}x_{ab}\left(5 - 2\alpha_{ab}R_{ab}^2\right) \qquad (S6.1)$$

$$T_{(ax)(by)} = \frac{1}{\sqrt{\alpha_a\alpha_b}}\frac{\partial}{\partial X_a}\frac{\partial}{\partial Y_b}T_{ab} \qquad (S6.2)$$

$$V_{(ax)bc} = \frac{1}{\sqrt{\alpha_a}}\frac{\partial}{\partial X_a}V_{abc} \qquad (S6.3)$$

$$V_{(ax)(by)c} = \frac{1}{\sqrt{\alpha_a\alpha_b}}\frac{\partial}{\partial X_a}\frac{\partial}{\partial Y_b}V_{abc} \qquad (S6.4)$$

and on for all other *X*, *Y*, *Z* combinations. After carrying out the differentiation, the special cases of coinciding nuclei, *a* = *b*, *a* = *c* , *b* = *c* and *a* = *b* = *c*, are readily derived.

S.3 Definition of a body-fixed coordinate system for density matrix parameterization

In this section we demonstrate the use of an inertia matrix in constructing a uniquely defined body fixed coordinate system. To begin, a 3x3 matrix is constructed using the nuclear positions $\mathbf{r}_k$ and nuclear weights w_k,

$$I_{\alpha\beta} = \sum_{k=1}^{N} w_k \left(|\mathbf{r}_k - \mathbf{r}_0|^2 \delta_{\alpha\beta} - (\alpha_k - \alpha_0)(\beta_k - \beta_0) \right) \qquad (S7)$$

where the index k runs over the N atoms with $\alpha,\beta = x,y,z$, and where the origin is defined in the usual way as

$$\mathbf{r}_0 = \frac{1}{W} \sum_{k=1}^{N} w_k \mathbf{r}_k \qquad (S8)$$

with $W = \sum_{k=1}^{N} w_k$. We presently set $w_k = 1$ which amounts to treating the nuclei as indistinguishable (Euclidean) points, although using nuclear charges has been seen as an equally valid approach in the calculations. On the other hand, using nuclear masses for w_k leads to an unphysical dependence of the basis and therefore the density matrix on isotopic substitutions and is therefore undesirable.

Diagonalization of $\mathbf{I}$ produces an orthogonal body-fixed (BF) basis $\hat{\mathbf{e}}_1$, $\hat{\mathbf{e}}_2$, $\hat{\mathbf{e}}_3$ ordered by the increasing eigenvalue. It is spatially unique for a given geometrical configuration but with the directions of the basis vectors determined only up to a sign. This ambiguity is problematic when density elements of the *sx*, *xy*, *etc* sort need to be calculated. A sign flip in $\hat{\mathbf{e}}_i$ leads to a flip, i.e. a discontinuity, in the corresponding density matrix element resulting in a discontinuous PES and its derivatives.

To address this issue, we adopt a convention by which a unique phase for the BF vectors is determined. By analogy with a dipole moment, a vector **D** is constructed relative to the above defined origin and using configuration dependent nuclear "charges"

$$\mathbf{D} = \sum_{k=1}^{N} \rho_k (\mathbf{r}_k - \mathbf{r}_0) \qquad (S9)$$

Examination of various models in a course of preliminary calculations suggested that $\rho_k = \gamma\ |\mathbf{r}_k - \mathbf{r}_0|^2$ is one of the simplest and most effective choices which guarantees a unique and non-zero vector **D** for a generic configuration and with permutational symmetry manifestly enforced, the latter property being easily checked by inspection. (It needs to be mentioned that in very special and extremely rare high-symmetry cases where **D** = 0 we assume $D_\alpha = |\hat{\mathbf{e}}_{1,\alpha}|^2$ for $\alpha = x,y,z$.) We also note that unlike a standard dipole moment vector, **D** is constructed to be invariant under translation which is a very important property in present derivation. Having constructed **D**, the phases of the BF axes are determined by the following convention: $\hat{\mathbf{e}}_1 \cdot \mathbf{D} > 0$, $\hat{\mathbf{e}}_2 \cdot \mathbf{D} > 0$, $\hat{\mathbf{e}}_3 = \hat{\mathbf{e}}_1 \times \hat{\mathbf{e}}_2$. Thus defined BF coordinate system is guaranteed to preserve invariance of the energy with respect to translations, rigid-body rotations and permutations of identical nuclei. Numerous tests of these cases have been done on a variety of molecules to verify this proposition numerically.

The fitting procedure involves the following steps:

(1) the training set coordinates (with corresponding energies) are taken from a trajectory propagation, Monte Carlo simulation or any other structure generator and transformed into the BF frame;

(2) the transformed set is used to construct the matrix equation for linear regression analysis and recovery of fitting coefficients c_i^{BF};

(3) to recover the energy of an arbitrary input geometry, the coefficients c_i^{BF} are used to express the density matrix **P** in the BF coordinate system which requires a prior rotation of the input geometry into the BF frame.

(4) The rotational covariance of the energy gradient is also properly described by the above procedure. Namely, the gradient in the input (space fixed (SF)) frame is first calculated analytically (or numerically) in the BF frame as $\vec{\nabla}E^{\mathrm{BF}}$ and then transformed back into the SF frame using

$$\left(\vec{\nabla}_i E\right)^{\mathrm{SF}} = \sum_{j=1}^{3} \left[\left(\vec{\nabla}_i E\right)^{\mathrm{BF}} \cdot \hat{\mathbf{e}}_j\right] \hat{\mathbf{e}}_j \qquad (S10)$$

for atom i, a protocol that one would employ in a molecular dynamics simulation. Similar transformations also work for higher order energy derivatives.

S.4 C_3 training and testing data

Following our previous approach of optimizing the linear parameters separately from the non-linear ones, here we perform a linear regression calculation for a set of the three exponents chosen on a grid: 1-dimensional for PIP and three-dimensional for CHM_s and CHM_sp models.

Table S1. C_3 training and testing data for the linear singlet. The training set **TR** contains 5001 trajectory points 2 fs apart propagated at ZPE (1726 cm^{-1}). **TEST** contains different 5000 trajectory points 2 fs apart from the same trajectory. **TEST-G** is the gradient data at the points of **TEST**.

	**PIP**	**term**	**TR**	**TEST**	**TEST-G**
PIP[1]	1	2	425 (1.0)	425	23496
CHM_s[1]	1	4	44 (6.0, 2.0, 0.3)	44	2679
CHM_sp[1]	1	28	0.5 (2.5, 2.0, 0.5)	1.5	103
PIP[2]	3	4	422 (1.2)	422	23744
CHM_s[2]	3	10	2.6 (2.5, 2.5, 1.2)	2.6	380
CHM_sp[2]	3	82	0.07 (2.0, 5.0, 0.9)	0.09	10
PIP[3]	6	7	39 (1.0)	39	4734
CHM_s[3]	6	19	0.4 (2.0, 2.0, 0.6)	0.4	64
PIP[4]	10	11	5.6 (4.0)	5.6	916
PIP[5]	15	16	1.3 (3.4)	1.3	184
PIP[6]	22	23	0.3 (2.0)	0.3	83

S.5 C_4 training and testing data

Table S2. C_4 training and testing data for the linear triplet. The training set **TR** contains 5001 trajectory points 2 fs apart propagated at ZPE (2848 cm^{-1}). **TEST** contains different 5000 trajectory points 2 fs apart from the same trajectory. **TEST-G** is the gradient data at the points of **TEST**.

	**PIP**	**term**	**TR**	**TEST**	**TEST-G**
PIP[1]	1	2	526 (5.0)	526	20839
CHM_s[1]	1	4	267 (6.5, 2.5, 0.3)	267	10789
CHM_sp[1]	1	28	35 (2.5, 4.5, 0.2)	35	2627
PIP[2]	4	5	297 (5.0)	297	20853
CHM_s[2]	4	13	12 (4.0, 3.5, 1.5)	12	960
CHM_sp[2]	4	109	0.2 (2.5, 3.0, 0.5)	0.2	20
PIP[3]	10	11	14 (1.2)	14	580
CHM_s[3]	10	31	1.5 (6.5, 3.0, 0.5)	1.5	91
PIP[4]	21	22	2.4 (3.4)	2.4	190
CHM_s[4]	21	64	0.4 (3.5, 2.0, 0.2)	0.4	27
PIP[5]	39	40	1.0 (2.6)	1.0	66

S.6 C_5 training and testing data

Table S3. C_5 training and testing data for the linear singlet. The training set **TR** contains 5001 trajectory points 2 fs apart propagated at ZPE (4176 cm^{-1}). **TEST** contains different 5000 trajectory points 2 fs apart from the same trajectory. **TEST-G** is the gradient data at the points of **TEST**.

	**PIP**	**term**	**TR**	**TEST**	**TEST-G**
PIP[1]	1	2	663 (5.0)	663	26055
CHM_s[1]	1	4	404 (6.5, 2.0, 0.3)	404	10609
CHM_sp[1]	1	28	71 (6.5, 2.0, 0.4)	71	3680
PIP[2]	4	5	607 (5.0)	607	26367
CHM_s[2]	4	13	56 (4.5, 5.5, 0.2)	56	3733
CHM_sp[2]	4	109	2.4 (3.5, 2.5, 0.1)	2.4	173
PIP[3]	11	12	103 (2.6)	103	4438
CHM_s[3]	11	34	4.6 (3.5, 2.5, 0.7)	4.6	232
CHM_sp[3]	11	298	0.2 (6.0, 2.5, 1.2)	0.2	23
PIP[4]	28	29	8.5 (1.4)	8.5	544
CHM_s[4]	28	85	0.8 (5.0, 3.0, 0.6)	0.8	55
PIP[5]	63	64	1.8 (2.4)	1.8	108

S.7 C_6 training and testing data

Table S4A. C_6 training and testing data for the cyclic singlet. The training set **TR** contains 5001 trajectory points 2 fs apart propagated at ZPE (6006 cm^{-1}). **TEST** contains different 5000 trajectory points 2 fs apart from the same trajectory. **TEST-G** is the gradient data at the points of **TEST**.

	**PIP**	**term**	**TR**	**TEST**	**TEST-G**
PIP[1]	1	2	852 (5.0)	852	25097
CHM_s[1]	1	4	539 (6.0, 2.0, 0.3)	539	14620
CHM_sp[1]	1	28	210 (4.5, 2.5, 0.3)	210	8742
PIP[2]	4	5	558 (5.0)	558	25856
CHM_s[2]	4	13	110 (3.0, 4.0, 0.2)	110	3390
CHM_sp[2]	4	109	18 (5.5, 6.0, 1.5)	18	951
PIP[3]	12	13	261 (4.2)	261	8172
CHM_s[3]	12	37	29 (4.0, 3.5, 0.2)	29	1294
CHM_sp[3]	12	325	3.5 (2.5, 2.0, 1.5)	3.5	214
PIP[4]	33	34	41 (3.6)	41	1413
CHM_s[4]	33	100	7.9 (3.0, 2.5, 0.2)	7.9	341
CHM_sp[4]	33	892	1.0 (2.0, 2.0, 1.5)	1.0	69
PIP[5]	85	86	11 (4.6)	11	398
CHM_s[5]	85	256	1.9 (2.5, 2.0, 1.2)	1.9	91
CHM_sp[5]	85	2296	0.4 (2.0, 2.5, 1.5)	0.4	36

Table S4B. C_6 training and testing data for the linear triplet. The training set **TR** contains 5001 trajectory points 2 fs apart propagated at ZPE (5121 cm^{-1}). **TEST** contains different 5000 trajectory points 2 fs apart from the same trajectory. **TEST-G** is the gradient data at the points of **TEST**.

	**PIP**	**term**	**TR**	**TEST**	**TEST-G**
PIP[1]	1	2	774 (1.0)	774	19254
CHM_s[1]	1	4	611 (6.5, 2.0, 0.3)	611	7557
CHM_sp[1]	1	28	102 (6.5, 2.0, 0.4)	102	4310
PIP[2]	4	5	535 (2.6)	534	19924
CHM_s[2]	4	13	124 (4.5, 2.0, 0.1)	124	4212
CHM_sp[2]	4	109	13 (6.5, 2.0, 0.7)	13	572
PIP[3]	12	13	58 (2.0)	58	1885
CHM_s[3]	12	37	19 (4.5, 3.0, 1.0)	19	605
CHM_sp[3]	12	325	2.0 (6.5, 2.0, 0.2)	2.0	130
PIP[4]	33	34	23 (2.6)	23	1182
CHM_s[4]	33	100	5.0 (5.0, 3.5, 0.1)	5.0	233
CHM_sp[4]	33	892	0.6 (2.0, 4.5, 0.1)	0.6	50
PIP[5]	85	86	8.5 (4.4)	8.5	439
CHM_s[5]	85	256	1.5 (5.0, 2.5, 0.1)	1.5	97
CHM_sp[5]	85	2296	0.2 (2.0, 3.0, 0.1)	0.2	37

S.8 C_7 training and testing data

Table S5. C_7 training and testing data for the linear singlet. The training set **TR** contains 5001 trajectory points 2 fs apart propagated at ZPE (6433 cm^{-1}). **TEST** contains different 5000 trajectory points 2 fs apart from the same trajectory. **TEST-G** is the gradient data at the points of **TEST**.

	**PIP**	**term**	**TR**	**TEST**	**TEST-G**
PIP[1]	1	2	835 (5.0)	834	27138
CHM_s[1]	1	4	1169 (6.5, 2.0, 0.3)	1169	25626
CHM_sp[1]	1	28	401 (6.5, 2.0, 0.4)	401	9136
PIP[2]	4	5	593 (2.4)	592	27960
CHM_s[2]	4	13	233 (6.5, 2.0, 0.1)	233	5855
CHM_sp[2]	4	109	39 (6.5, 2.0, 0.6)	39	1587
PIP[3]	12	13	340 (2.6)	340	5500
CHM_s[3]	12	37	60 (5.0, 3.0, 0.9)	60	2339
CHM_sp[3]	12	325	6.7 (6.5, 2.0, 0.1)	6.6	307
PIP[4]	34	35	83 (3.0)	83	3889
CHM_s[4]	34	103	10 (6.0, 3.0, 0.2)	10	400
CHM_sp[4]	34	919	1.8 (5.0, 3.5, 0.2)	1.8	97
PIP[5]	94	95	12 (3.6)	12	460
CHM_s[5]	94	283	3.3 (5.0, 3.0, 0.2)	3.1	140
CHM_sp[5]	94	2539	0.9 (2.0, 4.5, 0.1)	1.1	128

S.9 C_8 training and testing data

Table S6. C_8 training and testing data for the linear triplet. The training set **TR** contains 5001 trajectory points 2 fs apart propagated at ZPE (7326 cm^{-1}). **TEST** contains different 5000 trajectory points 2 fs apart from the same trajectory. **TEST-G** is the gradient data at the points of **TEST**.

	**PIP**	**term**	**TR**	**TEST**	**TEST-G**
PIP[1]	1	2	871 (5.0)	871	25332
CHM_s[1]	1	4	984 (6.5, 2.0, 0.3)	984	31040
CHM_sp[1]	1	28	372 (6.5, 2.0, 0.4)	372	11925
PIP[2]	4	5	574 (2.4)	574	26693
CHM_s[2]	4	13	322 (6.5, 2.0, 0.2)	322	6648
CHM_sp[2]	4	109	50 (6.5, 2.0, 0.6)	50	1642
PIP[3]	12	13	184 (2.0)	184	5070
CHM_s[3]	12	37	55 (5.0, 3.0, 1.0)	55	1737
CHM_sp[3]	12	325	12 (5.0, 2.5, 0.2)	12	474
PIP[4]	35	36	57 (1.6)	57	1861
CHM_s[4]	35	106	18 (6.0, 2.0, 0.2)	18	562
CHM_sp[4]	35	946	4.8 (4.5, 3.5, 0.1)	4.9	238
PIP[5*]	43	44	50 (1.8)	50	1493
CHM_s[5*]	43	130	15 (6.5, 3.0, 0.2)	15	525
CHM_sp[5*]	43	1162	4.5 (3.0, 3.5, 0.1)	4.6	236

S.10 C_9 training and testing data

Table S7. C_9 training and testing data for the linear singlet. The training set **TR** contains 5001 trajectory points 2 fs apart propagated at ZPE (8559 cm^{-1}). **TEST** contains different 5000 trajectory points 2 fs apart from the same trajectory. **TEST-G** is the gradient data at the points of **TEST**.

	**PIP**	**term**	**TR**	**TEST**	**TEST-G**
PIP[1]	1	2	941 (5.0)	940	21550
CHM_s[1]	1	4	1226 (6.5, 2.0, .3)	1226	36557
CHM_sp[1]	1	28	628 (6.5, 2.0, 1.5)	628	24505
PIP[2]	4	5	649 (2.4)	648	21470
CHM_s[2]	4	13	555 (6.5, 2.5, .5)	555	20759
CHM_sp[2]	4	109	104 (6.0, 2.0, .7)	104	3644
PIP[3]	12	13	376 (2.2)	376	6608
CHM_s[3]	12	37	173 (4.5, 3.0, .2)	173	6416
CHM_sp[3]	12	325	26 (5.0, 2.0, .3)	26	1326
PIP[4]	35	36	216 (1.4)	216	6600
CHM_s[4]	35	106	42 (2.5, 6.0, 0.2)	42	2344
CHM_sp[4]	35	946	6 (3.5, 6.0, .1)	6	408
PIP[5*]	43	44	168 (1.2)	168	7798
CHM_s[5*]	43	130	30 (4.0, 2.0, .2)	30	1514
CHM_sp[5*]	43	1162	7 (4.5, 2.0, .1)	7	401

S.11 C_{10} training and testing data

Table S8A. C_{10} training and testing data for cyclic singlet. The training set **TR** contains 5001 trajectory points 2 fs apart propagated at the ZPE (10499 cm^{-1}). **TEST** contains different 5000 trajectory points 2 fs apart from the same trajectory. **TEST-G** is the gradient data at the points of **TEST**.

	PIP	term	TR	TEST	TEST-G
PIP[1]	1	2	1102 (1.0)	1101	40197
CHM_s[1]	1	4	1262 (6.5, 2.0, 0.3)	1262	35959
CHM_sp[1]	1	28	639 (6.5, 2.0, 1.5)	638	18899
PIP[2]	4	5	950 (1.0)	949	28684
CHM_s[2]	4	13	478 (3.5, 2.0, 0.6)	478	12639
CHM_sp[2]	4	109	288 (3.0, 2.0, 0.1)	288	9603
PIP[3]	12	13	509 (2.6)	509	14405
CHM_s[3]	12	37	270 (2.5, 2.0, 0.1)	270	6975
CHM_sp[3]	12	325	100 (2.0, 2.5, 0.1)	100	3797
PIP[4]	35	36	345 (1.0)	345	8218
CHM_s[4]	35	106	104 (2.5, 2.0, 0.3)	104	2658
CHM_sp[4]	35	946	26 (3.5, 2.0, 0.1)	27	1575

Table S8B. C_{10} training and testing data for linear triplet. The training set **TR** contains 5001 trajectory points 2 fs apart propagated at ZPE (9395 cm^{-1}). **TEST** contains different 5000 trajectory points 2 fs apart from the same trajectory. **TEST-G** is the gradient data at the points of **TEST**.

	**PIP**	**term**	**TR**	**TEST**	**TEST-G**
PIP[1]	1	2	956 (5.0)	955	34521
CHM_s[1]	1	4	1479 (6.5, 2.0, 0.3)	1479	28292
CHM_sp[1]	1	28	818 (3.5, 4.5, 0.2)	818	18656
PIP[2]	4	5	735 (2.8)	735	36054
CHM_s[2]	4	13	562 (6.0, 2.0, 0.1)	561	9697
CHM_sp[2]	4	109	104 (3.0, 6.5, 0.4)	104	3724
PIP[3]	12	13	254 (2.2)	254	7356
CHM_s[3]	12	37	158 (2.0, 6.5, 1.0)	158	4358
CHM_sp[3]	12	325	28 (6.0, 4.5, 0.1)	28	998
PIP[4]	35	36	122 (1.6)	122	4482
CHM_s[4]	35	106	35 (2.0, 6.5, 0.1)	35	1091
CHM_sp[4]	35	946	10 (5.0, 2.0, 0.1)	10	524

S.12 Electronic structure and configuration data for C_6 and C_{10}

Table S9A: B3LYP/aug-cc-pVTZ(*spd*) Mulliken population fractions in % at the linear and cyclic geometries of C_6 for the singlet (M1) and triplet electronic states (M3).

Isomer	*s*	*p*	*d*
D_{3h}-cyclic-M1	52.92	44.80	2.28
C_{2v}-cyclic-M3	55.63	42.68	1.69
$D_{\infty h}$-linear-M1	49.09	49.38	1.55
$D_{\infty h}$-linear-M3	48.98	49.49	1.53

Table S9B: B3LYP/aug-cc-pVTZ(*spd*) Mulliken population fractions in % at the linear and cyclic geometries of C_{10} for the singlet (M1) and triplet electronic states (M3).

Isomer	*s*	*p*	*d*
D_{5h}-cyclic-M1	47.38	49.37	3.25
C_{2v}-cyclic-M3	48.39	48.77	2.84
$D_{\infty h}$-linear-M1	29.86	67.46	2.68
$D_{\infty h}$-linear-M3	29.78	67.54	2.68

Table 10A: Stationary points of C_6 calculated with B3LYP/aug-cc-pVTZ(*spd*).

Stationary points	Label	Imag freq	Energy	Enery+ZPE	Energy diff	ZPE diff	ZPE
Units		cm^{-1}	hartree	hartree	cm^{-1}	cm^{-1}	eV
TS-Lin-ring-M3	C_1	-453	-228.2200294	-228.198237	24168	23882	2.96
TS-Lin-ring-M1	C_1	-350	-228.2103252	-228.188452	26298	26029	3.23
Min-Linear-M3	$D_{\infty h}$	min0	-228.3301484	-228.307050	0	0	0.00
Min-Linear-M1	$D_{\infty h}$	min2	-228.3086657	-228.285566	4715	4715	0.58
Min-Ring-M1	D_{3h}	min1	-228.3209871	-228.293623	2011	2947	0.37
TS-Ring-M1	D_{3d}	-517	-228.2981708	-228.272496	7018	7584	0.94
Min-Ring-M3	C_{2v}	min3	-228.2752357	-228.250802	12052	12345	1.53
TS-Ring-M3	C_s	-402	-228.2609738	-228.239043	15182	14926	1.85
TS1-Ring-M3	C_{2v}	-451	-228.2714646	-228.248293	12880	12896	1.60

Table 10B: Stationary points of C_{10} calculated with B3LYP/aug-cc-pVTZ(*spd*).

Stationary points	Label	Imag freq	Energy	Enery+ZPE	Energy diff	ZPE diff	ZPE
Units		cm^{-1}	hartree	hartree	cm^{-1}	cm^{-1}	eV
TS-Lin-ring-M3	C_1	-278	-380.604644	-380.563415	39147	37695	4.67
TS-Lin-ring-M1	C_1	-176	-380.599027	-380.557075	40380	39086	4.85
Min-Linear-M3	$D_{\infty h}$	min2	-380.700327	-380.657520	18147	17041	2.11
Min-Linear-M1	$D_{\infty h}$	min3	-380.685673	-380.642822	21363	20267	2.51
Min-Ring-M1	D_{5h}	min0	-380.783012	-380.735166	0	0	0.00
TS-Ring-M1	D_{10h}	-415	-380.775965	-380.730398	1547	1046	0.13
Min-Ring-M3	C_{2v}	min1	-380.708243	-380.663232	16410	15788	1.96
TS-Ring-M3	D_{2h}	-158	-380.707850	-380.663635	16496	15699	1.95

There is a small D_{2h} barrier, ~90 cm^{-1}, to C_{2v} - C_{2v} pseudoinversion of the cyclic triplet C_{10}, calculated at B3LYP/aug-cc-pVTZ(*spd*) level, which completely disappears upon including the ZPE. Calculations using the cc-pVQZ basis set yield the triplet C_{10} cyclic D_{2h} barrier even closer in energy to the C_{2v} one, within ~30 cm^{-1}, therefore we presume that the vibrationally averaged triplet C_{10} configuration is of the D_{2h} geometry.

We also examine isomerization pathways connecting cyclic and linear configurations for both singlet and triplet electronic states of C_{10}. Previous DFT studies at the PBE/Def2TZVPP level of theory (see main text for details) reported only linear-cyclic isomerization for the singlet state. The transition state for the cyclic-linear isomerization, i.e., the ring opening process, of the C_{10} singlet state is much higher than that for the triplet state. The cyclic singlet C_{10} is lower in energy

than the cyclic triplet C_{10} state, while the linear singlet C_{10} is higher in energy than the linear triplet C_{10}.

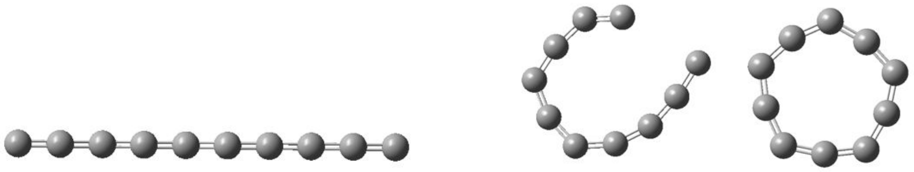

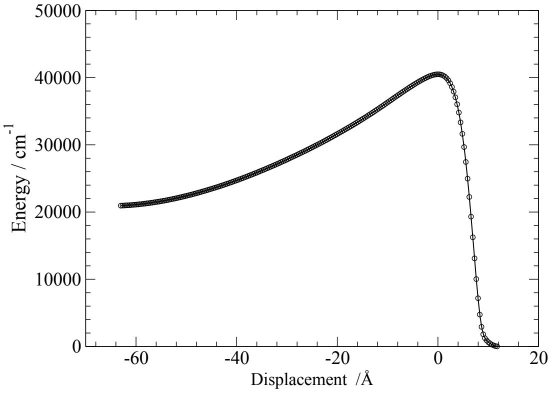

Figure S1: B3LYP/aug-cc-pVTZ(*spd*) IRC pathway connecting the linear and cyclic configurations of C_{10} in the singlet electronic state. The zero of energy corresponds to the cyclic D_{5h} configuration. The C-C distance between the terminal carbons in the TS is 2.914 Å.

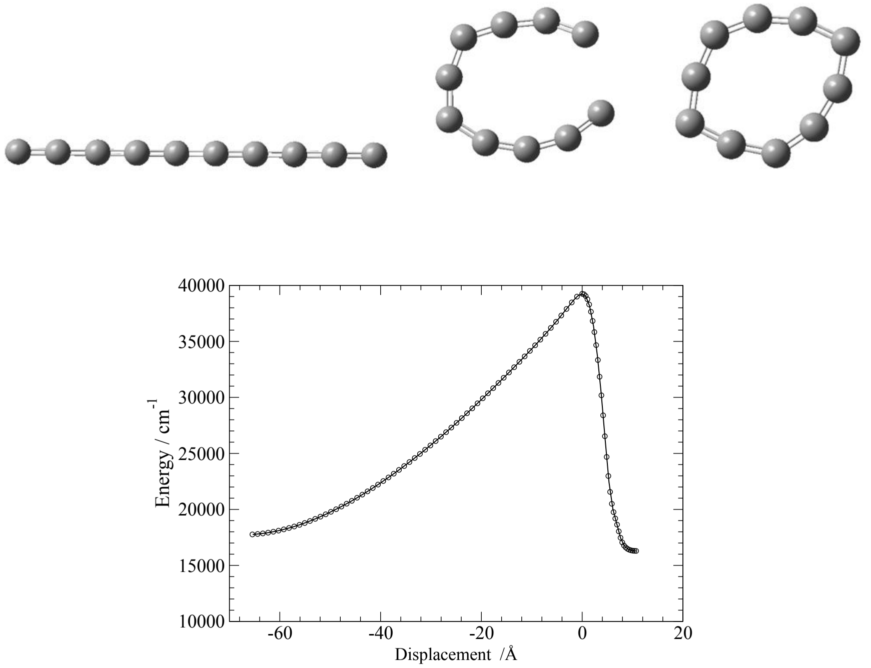

Figure S2: B3LYP/aug-cc-pVTZ(*spd*) IRC pathway connecting the linear and cyclic configurations of C_{10} in the triplet electronic state. The zero of energy corresponds to the cyclic D_{5h} configuration of the singlet. The C-C distance between the terminal carbons in the TS is 2.520 Å.

Table S11A. XYZ (Å) of TS-Lin-ring-M1 @ B3LYP/aug-cc-pVTZ(*spd*)

C	-1.864076	-1.022553	-0.047037
C	-0.999414	-1.953478	0.100153
C	0.295407	-1.884328	0.078056
C	-2.330581	0.183389	-0.085743
C	1.428325	-1.313711	0.032309
C	-1.630774	1.254536	-0.126563
C	-0.691154	2.149069	-0.056847
C	2.287353	-0.355193	-0.042851
C	2.978806	0.732952	-0.196695
C	0.526108	2.209316	0.345217

Table S11B. XYZ (Å) of TS-Lin-ring-M3 @ B3LYP/aug-cc-pVTZ(*spd*)

C	-2.084846	0.255428	-0.033345
C	-2.079202	-1.025728	0.038703
C	-0.985218	-1.729017	0.089919
C	-1.641233	1.465678	-0.098951
C	0.267114	-1.926514	0.076930
C	-0.417471	1.847596	-0.077216
C	0.866580	1.971628	-0.024621
C	1.515898	-1.587070	0.021173
C	2.518375	-0.836259	-0.295831
C	2.040001	1.564257	0.303239

S.13 C_{20} training and testing data

Table S12. C_{20} training and testing data. The training set **TR** contains 5001 trajectory points 2 fs apart propagated at ZPE/16 (1579 cm^{-1}). **TEST** contains different 5000 trajectory points 2 fs apart from the same trajectory, and **TEST-G** contains the gradient data at the points of **TEST**.

	**PIP**	**term**	**TR**	**TEST**	**TEST-G**
PIP[1]	1	2	107 (2.0)	107	12521
CHM_s[1]	1	4	473 (2.0, 3.5, 0.7)	473	36890
CHM_sp[1]	1	28	105 (4.5, 6.5, 1.3)	105	10790
PIP[2]	4	5	104 (1.0)	104	12054
CHM_s[2]	4	13	82 (2.5, 3.5, 0.6)	81	8339
CHM_sp[2]	4	109	41 (2.0, 3.0, 1.5)	41	3974
PIP[3]	12	13	95 (1.0)	95	10209
CHM_s[3]	12	37	58 (5.5, 3.5, 1.2)	58	4617
CHM_sp[3]	12	325	22 (2.0, 3.0, 1.4)	22	2449
PIP[4*]	29	30	68 (1.2)	68	6557
CHM_s[4*]	29	88	35 (3.0, 2.0, 0.6)	35	3263
CHM_sp[4*]	29	784	15 (2.0, 6.0, 1.3)	15	1834
PIP[4]	35	36	67 (1.4)	67	6502
CHM_s[4]	35	106	33 (3.0, 2.0, 1.5)	33	N/A
CHM_sp[4]	35	946	16 (2.0, 6.0, 0.2)	16	N/A

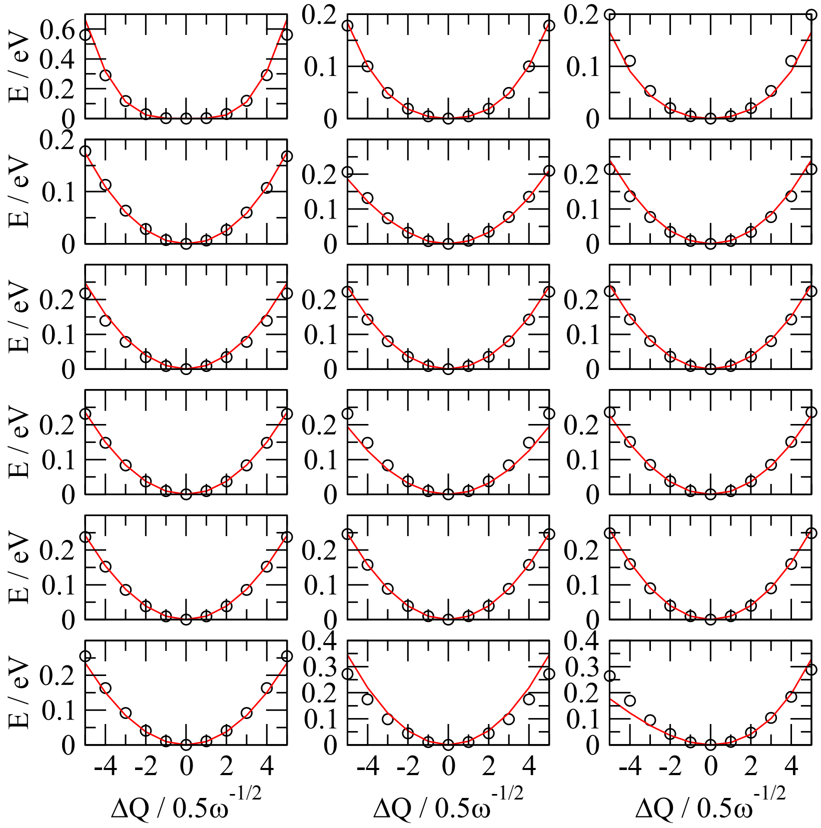

Figure S3. Normal mode cuts of the C_{20} potential for modes n = 1 - 18 (ordered left to right, top to bottom) along the reduced coordinate where ω is the corresponding harmonic frequency. The red curve is the **TR**-trained CHM_cp[4*] model, and the black open circles are the M06L/6-31G data.

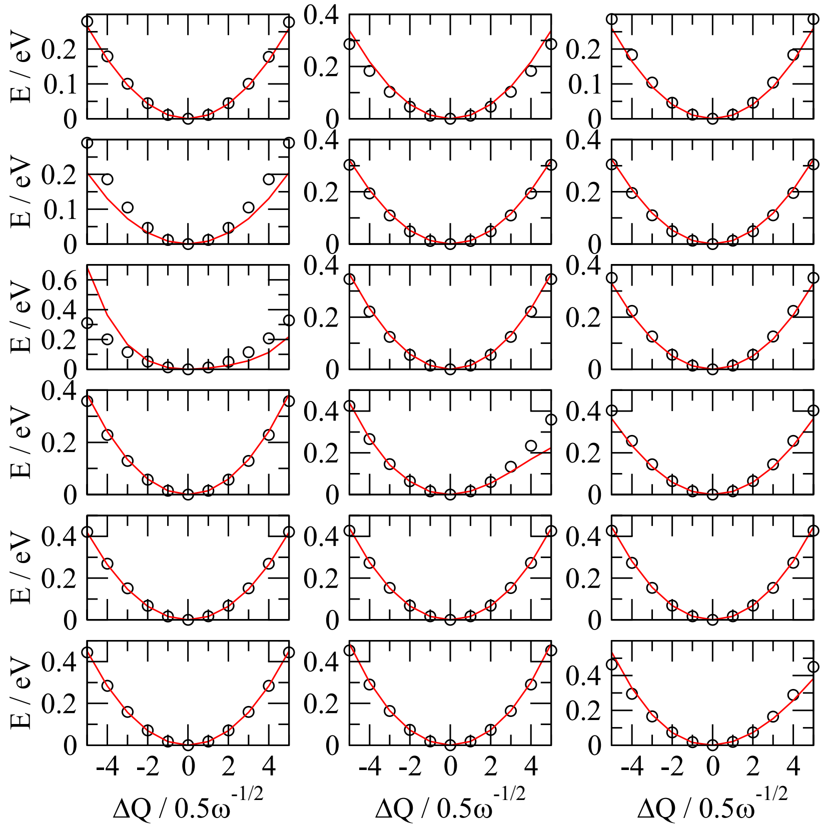

Figure S4. Normal mode cuts of the C_{20} potential for modes n = 19 - 36 (ordered left to right, top to bottom) along the reduced coordinate where ω is the corresponding harmonic frequency. The red curve is the **TR**-trained CHM_cp[4*] model, and the black open circles are the M06L/6-31G data.

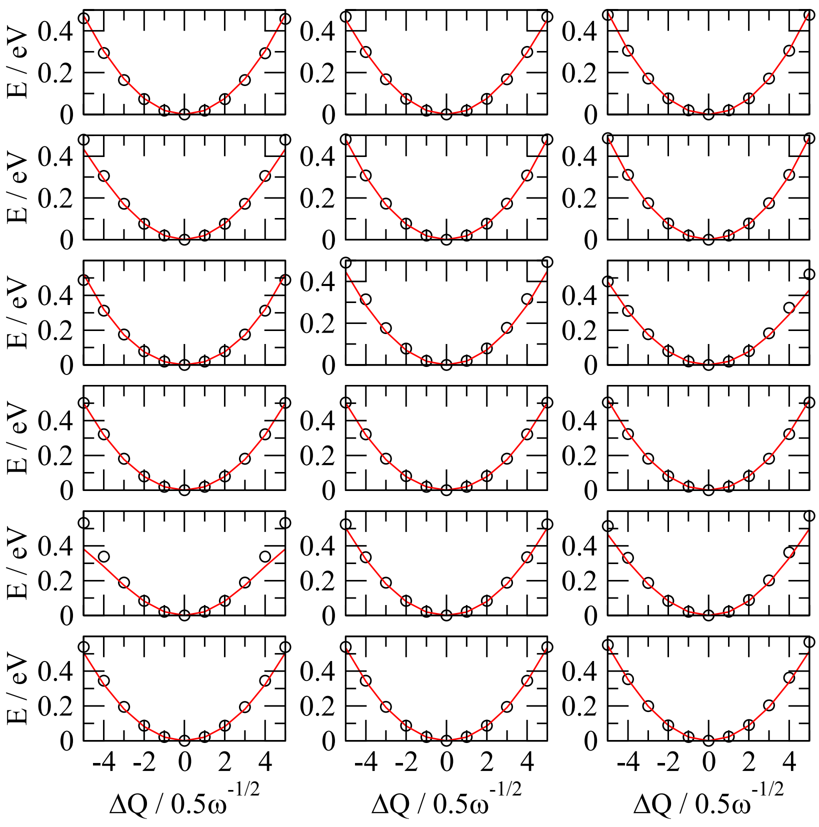

Figure S5. Normal mode cuts of the C_{20} potential for modes n = 37 - 54 (ordered left to right, top to bottom) along the reduced coordinate where ω is the corresponding harmonic frequency. The red curve is the **TR**-trained CHM_cp[4*] model, and the black open circles are the M06L/6-31G data.

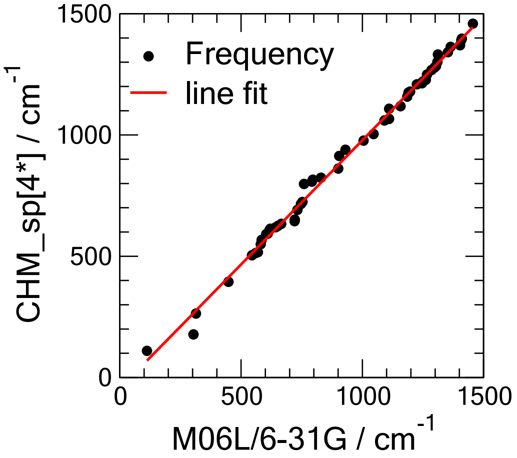

Figure S6. Comparison of the harmonic frequencies of the **TR**-trained CHM_sp[4*] model (y-axis) and the exact data (x-axis), (black dots). The linear fit (red line) is y = -45.127 + 1.0232 x.
